# Topography of the Dolomites modulates range dynamics of narrow endemic plants under climate change

**DOI:** 10.1038/s41598-022-05440-3

**Published:** 2022-01-26

**Authors:** Francesco Rota, Gabriele Casazza, Giulio Genova, Gabriele Midolo, Filippo Prosser, Alessio Bertolli, Thomas Wilhalm, Juri Nascimbene, Camilla Wellstein

**Affiliations:** 1 Faculty of Science and Technology, Free University of Bozen‐Bolzano, Bolzano, Italy; 2 Dipartimento di Scienze della terra, dell’Ambiente e della Vita, Università Di Genova, Corso Europa 26, 16132 Genova, Italy; 3 Fondazione Museo Civico Di Rovereto, Rovereto, Trento Italy; 4 Museum of Nature South Tyrol, Via Bottai 1, 39100 Bolzano, Italy; 5 BIOME Lab, Department of Biological, Geological and Environmental Sciences, Alma Mater Studiorum, University of Bologna, Bologna, Italy

**Keywords:** Biodiversity, Biogeography, Climate-change ecology, Conservation biology, Ecological modelling

## Abstract

Climate change is expected to threaten endemic plants in the Alps. In this context, the factors that may modulate species responses are rarely investigated at a local scale. We analyzed eight alpine narrow endemics of the Dolomites (southeastern Alps) under different predicted climate change scenarios at fine spatial resolutions. We tested possible differences in elevation, topographic heterogeneity and velocity of climate change among areas of gained, lost, or stable climatic habitat. The negative impact of climate change ranged from moderate to severe, depending on scenario and species. Generally, range loss occurred at the lowest elevations, while gained and stable areas were located at highest elevations. For six of the species, climate change velocity had higher values in stable and gained areas than in lost ones. Our findings support the role of topographic heterogeneity in maintaining climatic microrefugia, however, the peculiar topography of the Dolomites, characterized by high altitude plateaus, resulted in high climate change velocity in areas of projected future climatic suitability. Our study supports the usefulness of multiple predictors of spatio-temporal range dynamics for regional climate-adapted management and eventual assisted colonization planning to not overlook or overestimate the potential impact of climate change locally.

## Introduction

Global warming, driven by anomalous anthropogenic CO_2_ emission^1^, is one of the greatest threats to biodiversity^2,3^. Under a predicted future climate, the Alps will be severely affected by the end of the twenty-first century, especially in high elevation ranges^4^. Among mountain species, endemic plants are expected to be more affected by climate change due to their small niche-breadths, small population sizes, low genetic diversity, specific habitat requirements^5^ and low dispersal ability^6^. In particular, in the Alps^7–9^, endemic plants growing at high elevation are more sensitive to climate change than endemics growing at low elevation. Moreover, endemics restricted to the tops of not snow-cladded mountains and endemics limited by lithological (e.g. massive limestone) and/or pedological barriers are supposed to be most threatened since they may hardly shift their distributional range tracking new suitable areas^10^. Such characteristics of endemic plants call for an appropriate analysis of various factors that may modulate their responses to climate change, including elevation, topography and velocity of climate change.

In fact, to keep pace with global warming and avoid extinction, besides plasticity and evolutionary adaptation^11^, most mountain plant species are expected to shift their distributional range upward, causing a change of current vegetation communities and altering the equilibrium of high mountain ecosystems^12,13^. Species occurring at the highest altitudes may be extensively affected because they cannot shift their distributional range further upward^14^. Nevertheless, the high spatial heterogeneity that characterizes alpine landscapes^15^, may result in microclimatic variation, which in turn may buffer species against local extinction during climate changes^16–18^. In alpine environments, topography may affect species distribution indirectly through its correlation with temperature and precipitation, but also through landscape diversity and configuration as well as soil and water dynamics^19^. Consequently, a complex topography may favor the presence of microrefugia due to increased habitat diversity and niche space availability^20,21^. Microrefugia have increased the likelihood of species persistence during periods of environmental alterations (e.g., during past climatic fluctuations^22,23^). For these reasons, topographically complex alpine areas are considered a much ‘safer’ place to live under climate change than flat areas, which offer no short-distance escapes from new climatic conditions^24^.

Even if mountain topography buffered the effects of climate change on plants^25^, the poor ability to disperse of endemic plants^26^ can impede them from tracing the geographical shift in climatically suitable environments. For this reason, areas with a low rate of climate change may have favored^27^, and likely will favor, the persistence of endemic species^28^.

The Dolomites are one of the most relevant areas for plant biodiversity in the Alps^29^, being recognized as one of the richest centers of endemism and one of the main areas of glacial refugia^30,31^. Moreover, these mountains have unique geological and resulting topographical structures^32,33^ and are one of the most protected regions in the Alps, with ten natural parks and nine systems according to UNESCO within the World Heritage Sites^34^. In these areas, vegetation changes (i.e. increasing species numbers at high elevations and ‘thermophilization’) are currently taking place in the alpine zones^35,36^. However, as the Dolomites likely acted as refugia for cold adapted species during the last glaciations ensuring climatically stable areas, they might similarly enable species to survive future climate change in local refugia^28^.

Species distribution models (SDMs^37^) allow to predict the shift in suitable climatic conditions of species under environmental change^38^. However, because coarse resolution climate modeling approaches do not account for the importance of microclimatic conditions, they may underestimate the habitat suitability and overestimate the species’ rate of extinction^39^. For such reason, fine-scale modeling based on climatic downscaling techniques can be more effective in considering such microhabitat conditions^40^.

Using species distribution models, we analyzed the potential effects of climate change on eight plants endemic to the Dolomites under different climate change scenarios. Moreover, we tested whether areas where species are projected to lose, gain or maintain their suitable habitat differ in elevation, topographic heterogeneity and in velocity of climate change. First, we expected an extensive reduction of the distributional range of the endemics in the Dolomites, little balanced by gain of newly suitable areas. Secondly, we expected that endemics lose climatically suitable habitats at low elevation, in areas with low topographic heterogeneity and high climate change velocity, and that they maintain or gain suitable habitat at high elevation, in areas with high topographic heterogeneity and a low climate change velocity.

## Methods

### Study area and selected species

**Figure 1:**
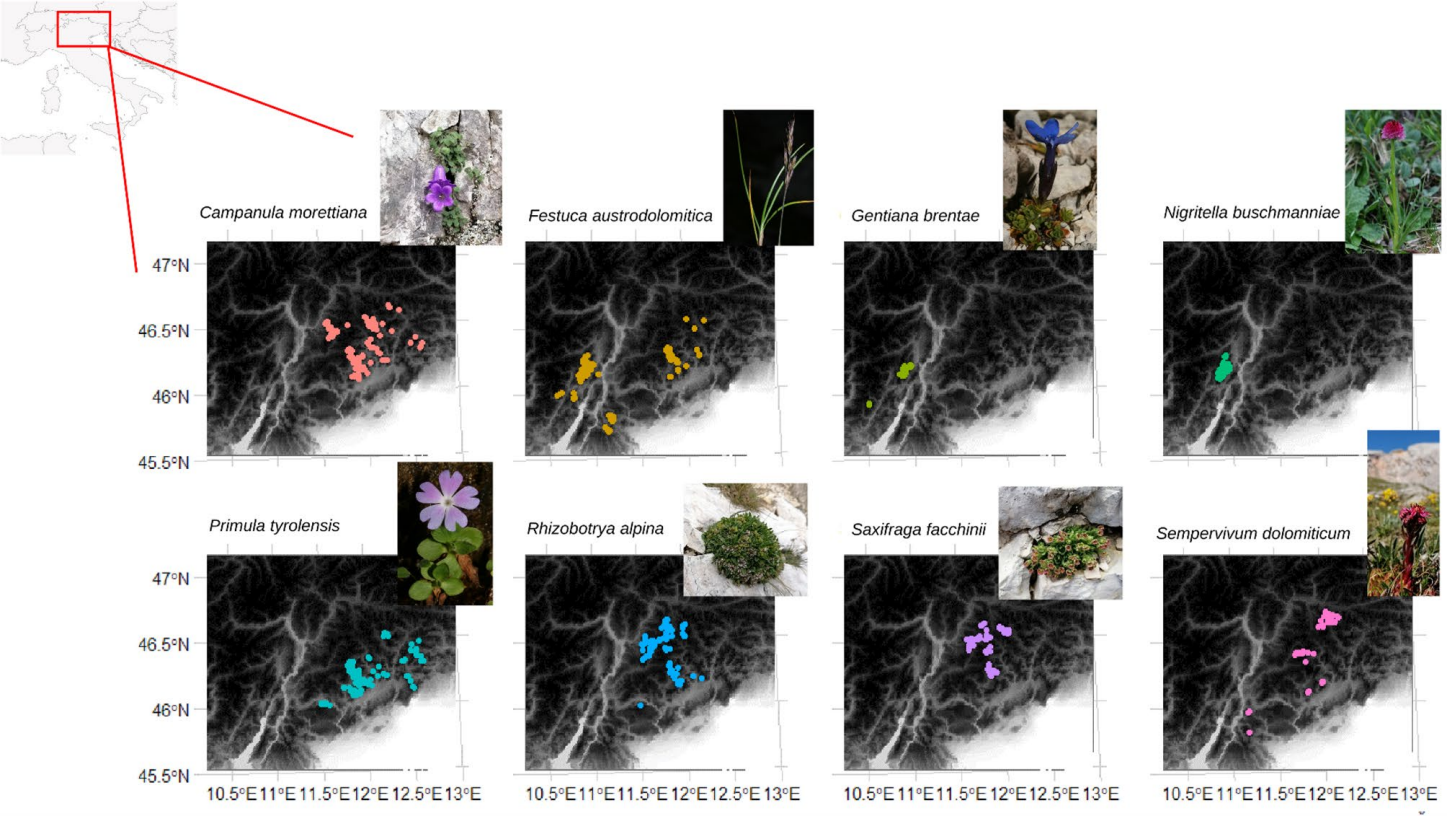
Study area location (Dolomites, south-eastern Alps, Italy) and species occurrences (colored dots) for the eight narrow endemic study species within their distribution range. Photo by Rota F. (*Campanula morettiana* Rchb. *Rhizobotrya alpina* Tausch, *Saxifraga facchinii* Koch and *Sempervivum dolomiticum* Facchini), photo by Prosser F. (*Festuca austrodolomitica* Pils and Prosser, *Gentiana brentae* Prosser and Bertolli, *Primula tyrolensis* Schott ex Rchb., *Nigritella buschmanniae* Teppner and Ster).

The study area includes the Dolomites and surrounding areas with a total area of about 8325 km^2^. In the last centuries, the field surveys carried out by several botanists provided an extensive documentation on the distribution of endemic plants^29,41–43^. In this study, we selected eight out of 25 endemic species with a restricted range present in the Dolomites^43^ (Table 1, S1; Fig. 1): *Campanula morettiana* Rchb. (Campanulaceae), *Festuca austrodolomitica* Pils and Prosser (Poaceae), *Gentiana brentae* Prosser and Bertolli (Gentianaceae), *Nigritella buschmanniae* Teppner and Ster (Orchidaceae), *Primula tyrolensis* Schott ex Rchb. (Primulaceae), *Rhizobotrya alpina* Tausch (Brassicaceae), *Saxifraga facchinii* Koch (Saxifragaceae) and *Sempervivum dolomiticum* Facchini (Crassulaceae).

**Table 1:** Life-history traits and conservation assessment for the eight studied species. For each species we reported the family, the life form (LF; H scap = scapose hemicryptophyte, H caesp = bushy hemicryptophyte, H ros = rosulate hemicryptophyte, Ch pulv = pulvinate chamaephyte, G bulb = bulb geophyte, Ch succ = succulent chamaephyte), the dispersal mode (DM), the inclusion in the Annex IV of the European Habitats Directive 92/43/EEC (HD; yes or no) and the IUCN category both at national level^45,46^ (It) and at regional level^42,47,48^ (Ve = Veneto, St = South-Tyrol and Tr = Trentino).

Species	Family	n	LF	DM	HD	IUCN category			
						It	Ve	St	Tr
*Campanula morettiana* Rchb	Campanulaceae	145	H scap	Boleochory (anemochory)	yes	LC	NT	EN	NT
*Festuca austrodolomitica* Pils and Prosser	Poaceae	161	H caesp	Pterometeorochory (anemochory)/Epizoochory (zoochory) for small mammals	no	LC	NT	NT	–
*Gentiana brentae* Prosser and Bertolli	Gentianaceae	72	H ros/Ch pulv	Boleochory (anemochory)	no	NT	–	–	NT
*Nigritella buschmanniae* Teppner and Ster	Orchidaceae	124	G bulb	Boleochory (anemochory)	no	NT	–	–	EN
*Primula tyrolensis* Schott ex Rchb	Primulaceae	147	H ros	Boleochory (anemochory)	no	LC	–	–	NT
*Rhizobotrya alpina* Tausch	Brassicaceae	112	H ros	Blastochory (autochory)	no	VU	VU	LC	NT
*Saxifraga facchinii* Koch	Saxifragaceae	93	H scap	Blastochory (autochory)	no	NT	EN	VU	NT
*Sempervivum dolomiticum* Facchini	Crassulaceae	49	Ch succ	Boleochory (autochory)	no	LC	VU	VU	VU

The species were selected based on their sub-alpine/alpine distribution and on the availability of more than 30 points of occurrence, which has been shown to provide reliable results in modeling studies^44^. Moreover, these species represent the taxonomic and ecological diversity of endemics in the alpine zone of the Dolomites belonging to eight families and growing in different habitats (i.e. screes, rocks, ridges and alpine grasslands).

All eight taxa are of conservation concern^45–48^ due to their narrow distribution range, rarity and high habitat specificity (Table 1). We obtained occurrences from public regional floristic databases and expert observations (Supplementary Table S1). We neither collected nor handled plants for our study. We discarded occurrences with uncertainty in taxonomic identification and inaccurate geographical position. To mitigate pseudo-replication of occurrences, we retained for each species only one occurrence per grid cell resampling the occurrence data at 50 m of resolution. In total, we included 939 occurrences, ranging from 49 to 161 occurrences per species.

### Environmental predictors

We downloaded all the 19 bioclimatic predictors for baseline (i.e. 1979–2013) and future (i.e. 2061–2080) climate at 30 arc sec (~ 1 km) spatial resolution from the CHELSA database^49^. We selected two future Representative Concentration Pathways (rcp’s) representing intermediate (rcp 4.5) and realistic (rcp 8.5) emission scenarios^50^. To account for variability in the forecasts, according to Sanderson et al.^51^, we used rcp’s projections from five non interdependent General Circulation Models (GCMs) representing physical processes in the atmosphere, ocean, cryosphere and land surface: ACCESS1-0, CESM1-CAM5, CNRM-CM5, MIROC-ESM, MPI-ESM-MR. To improve model’s transferability, we used the first two axes of a principal components analysis (PCA) as environmental variables for species distribution modeling. The PCA was calculated on the bioclimatic variables for baseline and for future climates pooled together; then the values of the first two axes of the PCA of each climate were separated to obtain the final response variables for each climate (one baseline and ten future). The PCA was performed using the R package factoextra^52^.

### Climate data downscaling

Because coarse scale models may underestimate the potential impacts of climate change on mountain plants not considering the microclimatic conditions, we statistically downscaled the climatic predictors at 50 × 50 m resolution. In short, we resampled through Nearest Neighbor a DEM at 50 m resolution, from the downloaded 25 m EU-DEM (https://www.eea.europa.eu/data-and-maps/data/eu-dem). We calculate slope and aspect, the latter as northness [*cos(aspect)*] and eastness [*sin(aspect)*], by using terrain() function in the R package “raster”. We included also an irradiation predictor (Direct Normal Irradiation, DNI [kWh/m^2^]) from https://globalsolaratlas.info/downloads/world. Then, we interpolated the PC1 and PC2 climatic predictors on the five topographical variables at 50 m (elevation, slope, northness, eastness, DNI) following the method of Geographic Weighted Regressions as in Lenoir et al.^53^.

### Species distribution models

To account for model-based uncertainties in the modelling process^54^, we used five SDM techniques implemented in the R package ‘biomod2’ v 3.3–7.1^55^. These five modeling techniques belong to three different model classes: two regression methods (Generalized Linear Modelling – GLM and Generalized Additive Modelling—GAM), two machine learning methods (Gradient Boosting Model – GBM and Random Forests – RF) and one classification method (Classification Tree Analysis—CTA). The number of pseudo-absences and replicate sets for each modeling technique was chosen according to the recommendations of Barbet-Massin et al.^56^ (Supplementary Table S2). For each pseudo-absence set, a split-sample cross-validation was repeated 10 times, using a random subset (30%) of the initial data set. We assessed model performance by using the area under the receiver operating characteristic curve (AUC)^57^ and the true skill statistic (TSS)^58^. As the choice of threshold may affect projection bias, we used three different thresholds performing equally or better than others^59,60^ to convert the continuous suitability maps into binary projections of species presence and absence: the threshold selection method based on (i) equal training sensitivity and specificity, (ii) maximizing training sensitivity and specificity, and (iii) minimizing the distance between the curve and the upper left corner of ROC plot. Finally, we generated a total of 165 binarized projections for each species: 15 for baseline climate (3 threshold * 5 algorithms) and 75 for each future climate rcp scenario (3 threshold * 5 algorithm * 5 GCMs). Then, we applied the majority consensus rule among the binarized projections: we considered a species as occurring in a cell if at least 50% of all the models predicted its occurrence there. Further, as the study species grow only on limestones and dolomitic rocks^42^, we used a geological layer obtained rasterizing at 50 m the geolithological map of Italy downloaded from the Geoportale Nazionale (http://www.pcn.minambiente.it/mattm/) to mask SDMs outputs (Supplementary Figure S1). All analyses were carried out on the Vienna Scientific Cluster (VSC) by using R (version R/3.6.2)^61^.

### Range analysis and maps

The percentage of overall projected range change (RC) in relation to the present-day predicted distribution was estimated using the formula RC = 100 * (RG – RL)/PR, where RG (range gain) is the number of grid cells projected to be not suitable under present climate but suitable under future climate, RL (range loss) is the number of grid cells projected to be suitable under present climate but not suitable under future climate and PR (present range) is the number of grid cells predicted suitable under baseline climatic scenario. A negative RC value indicates a loss in overall range, whereas a positive value indicates an increase in overall range size. The no-dispersal scenario was calculated as the percentage of range lost (percentage of RL), while the full-dispersal scenario was calculated as percentage of range changed (percentage of RC), as the result of the difference between RG and RL related to the present range (PR). For each species and scenario, mean and standard deviation values were calculated among each combination of algorithm, GCMs and thresholds. The full-dispersal scenario is likely to be unrealistic because it assumes that a species can colonize all locations without physiological, environmental or geographical limitations, while the no-dispersal scenario is likely to be more appropriate for poor dispersers as endemic species frequently are^26^ (Table 1).

### Climatic habitat-suitability relationship with elevation, topographic heterogeneity and climate change velocity

We explored the relationship among change in habitat suitability (loss or stability in the future) and elevation, topographic heterogeneity and climate change velocity (CCV). Elevation was assessed from the DEM at 50 m resolution (see above). The topographic heterogeneity was calculated with two different indices: (i) the Terrain Ruggedness Index (TRI)^62^ with “tri()” function of “spatialEco” package in R that estimates for the among-cells grid complexity and is calculated as the mean of the absolute differences between the value of a cell and the value of its eight surrounding cells, (ii) the intrinsic topographic complexity index (TCI) to account for within-cells grid complexity (following Irl et al.^63^), calculating the ratio between the 3D and 2D surface area, including the Tinitaly DEM at 10 m resolution (from http://tinitaly.pi.ingv.it/)^64^, with the following equation:

$$TCI = \frac{{\mathop \sum \nolimits_{{\left( {50\times50} \right)m}} \left( {{\raise0.7ex\hbox{${Area_{{\left( {10\times10} \right)m}} }$} \!\mathord{\left/ {\vphantom {{Area_{{\left( {10\times10} \right)m}} } {\cos (Slope_{{\left( {10\times10} \right)m}} )}}}\right.\kern-\nulldelimiterspace} \!\lower0.7ex\hbox{${\cos (Slope_{{\left( {10\times10} \right)m}} )}$}}} \right)}}{{Area_{{\left( {50x50} \right)m}} }}$$

The multivariate climate change velocity surface was calculated using the protocol described in Hamann et al.^65^. In short, climate change velocity was calculated as the minimum geographic distance to the nearest analogous climate, divided by the number of years between the baseline climate period and the future projection for both the intermediate (rcp 4.5) and the realistic (rcp 8.5) climate scenario. The CCV value is calculated as log_10_ of velocity (m*yr^-1^) multiplied by 100. We used the first two PCA axes calculated from the 19 bioclimatic variables and 120 bins of unique climates to define climate matches for each of the 5 GCMs, then we averaged them to obtain two scenarios of CCV (rcp 4.5 and rcp 8.5).

We assessed the correlation among the five variables (elevation, TCI, TRI, CCV rcp4.5, CCV rcp8.5) over the whole study area using the Pearson correlation coefficient implemented in R.

We created a matrix of each loss, gain and stable spatio-temporal category of difference of climatic habitat suitability between the baseline and the future predicted conditions for each species. We analyzed the relationship among habitat suitability and elevation, topographic heterogeneity and climate change velocity for each species using the Kruskal–Wallis test, with 0.05 significance. Subsequently, we tested the difference of elevation, topographic heterogeneity and climate change velocity among areas of climatic loss, stability or gain by using the post-hoc Mann–Whitney test (pairwaise.wilcox.test() R function) with 0.05 significance and “Bonferroni” correction^66^ for each species. To test for the robustness of these results and for pseudo replication issues, we run the analysis randomly sampling 10% and 1% of cells of each range change category. We repeated the randomization procedure 1000 times, then we counted how many times the Kruskal–Wallis test was significant (*p* value <  = 0.05). Second, we performed the previous procedure setting the maximum number of cells of each range change category to 1000.

## Results

### Overall patterns of change in habitat suitability

Under baseline climatic conditions, model evaluation showed a good model performance for the majority of modelling techniques in all analysed species (Supplementary Table S3). Considering each species’ response, range loss was different between the two future scenarios. In particular, the percentage of loss ranged from 51 to 79% under the rcp 4.5 scenario, while it was higher under the rcp 8.5 scenario ranging from 72 to 92% (Table 2, Table S4).

**Table 2:** Percentage of range change (RC), range loss (RL) and range gain (RG) under two future emission scenarios (intermediate rcp 4.5 and realistic rcp 8.5) for the eight study species.

Species	RCP 4.5 scenario			RCP 8.5 scenario		
	RC (%)	RL (%)	RG (%)	RC (%)	RL (%)	RG (%)
*Campanula morettiana*	− 55.98 (17.8)	− 56.29 (17.60)	0.31 (0.73)	− 79.4 (19.53)	− 79.72 (19.50)	0.32 (0.82)
*Festuca austrodolomitica*	− 65.29 (13.10)	− 67.88 (13.78)	2.56 (2.06)	− 82.76 (15.55)	− 85.59 (15.23)	2.83 (2.44)
*Gentiana brentae*	− 33.67 (11.06)	− 54.34 (21.58)	20.67 (20.05)	− 53.11 (25.48)	− 74.11 (25.27)	21.01 (21.22)
*Nigritella buschmanniae*	− 39.71 (9.63)	− 79.79 (16.16)	40.08 (18.59)	− 49.57 (19.56)	− 92.13 (10.94)	42.56 (25.67)
*Primula tyrolensis*	− 41.46 (16.96)	− 63.60 (14.37)	22.15 (6.10)	− 72.43 (20.49)	− 84.72 (13.76)	12.28 (8.23)
*Rhizobotrya alpina*	− 58.88 (16.82)	− 59.91 (16.54)	1.03 (1.37)	− 81.72 (19.11)	− 82.10 (18.66)	0.38 (0.73)
*Saxifraga facchinii*	− 67.85 (21.29)	− 75.52 (19.86)	7.67 (16.60)	− 59.29 (71.35)	− 87.59 (20.06)	28.29 (71.75)
*Sempervivum dolomiticum*	− 50.81 (27.56)	− 51.33 (27.38)	0.52 (1.02)	− 71.25 (30.59)	− 71.81 (29.57)	0.55 (1.34)

Standard deviation values are given in brackets

The habitat loss mainly occurred at lower elevations and at the geographical periphery of distributional range of each species (Supplementary Figures S3–S17). The percentage of range gain was low (< 10%) for *C. morettiana*, *F. austrodolomitica*, *R. alpina*, *S. dolomiticum* and *S. facchinii.* Differently, *G. brentae* and *P. tyrolensis* gained ca. 20% of range, while *N. buschmanniae* gained ca. 40% in the rcp 4.5 scenario. Under the rcp 8.5 scenario, the range gain was generally lower, and *C. morettiana*, *R. alpina* and *S. dolomiticum* did not show any range gain. Under the rcp 8.5 scenario, *S. facchinii* was projected to balance out the range loss with a high range gain (28%), albeit the standard deviation of the result was very high (71.75%). These values resulted in a high percentage of range change for all species. Despite a general range reduction, we projected a high extinction risk (i.e. range change from − 80 to − 100%) only for *R. alpina* and *F. austrodolomitica* under the rcp 8.5 scenario, even if *C. morettiana, P. tyrolensis* and *S. dolomiticum* showed values between -70 and -80%.

### The correlation among changes in climatic habitat suitability and elevation, topographic heterogeneity and climate change velocity

Elevation was correlated by 18% and 19% respectively with TCI and TRI and by 26% with the intermediate CCV, while by 49% with the realistic CCV. TCI and TRI showed a correlation of 65%. A low correlation was found for the topographic heterogeneity parameters with CCV, with a little increase in the realistic scenario (Table 3).

**Table 3:** Correlation matrix among the tested variables for habitat suitability: elevation, topographic heterogeneity (as TCI, topographical intrinsic complexity, and TRI, terrain ruggedness) and climate change velocity for both rcp 4.5 (CCV_45) and rcp 8.5 (CCV_85).

	Elevation	TCI	TRI	CCV_45	CCV_85
Elevation	1	0.18	0.19	0.26	0.49
TCI	0.18	1	0.66	0.02	0.08
TRI	0.19	0.66	1	0.00	0.06
CCV_45	0.26	0.02	0.00	1	0.74
CCV_85	0.49	0.08	0.06	0.74	1

**Figure 2:**
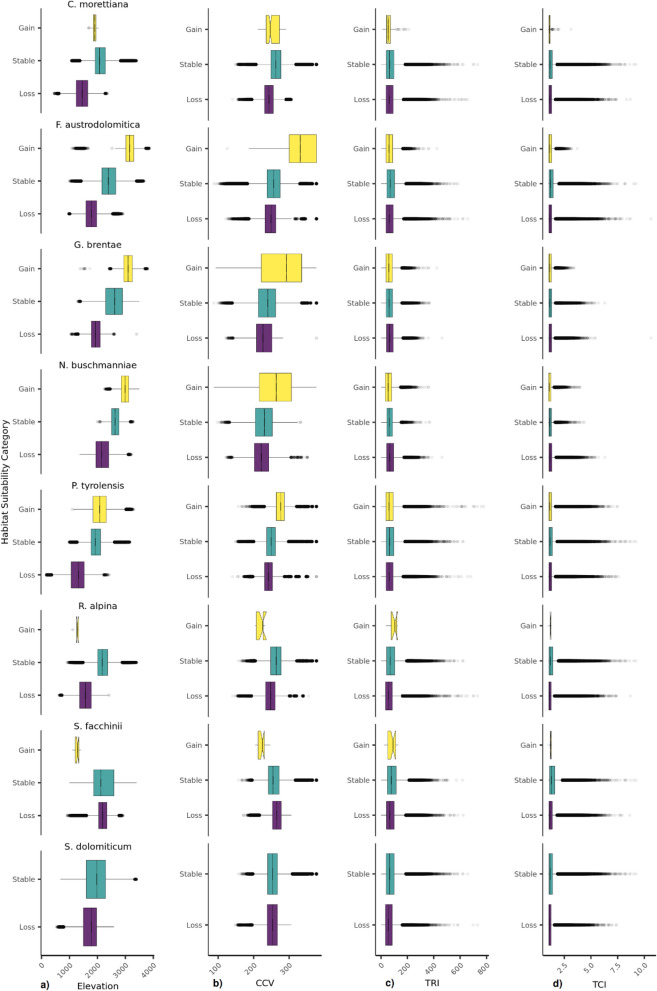
Boxplot for each category of habitat suitability (loss, stable, gain) for the intermediate rcp 4.5 scenario for (**a**) elevation (m a.s.l.), (**b**) climate change velocity (log10(m yr^−1^)*100), (**c**) topographical ruggedness index (TRI) and (**d**) topographical complexity index (TCI) for each of the eight study species. After the pairwise Mann–Whitney tests all the categories showed significant differences, with the following non-significant exceptions for the rcp 4.5 scenario: gained areas compared to lost and stable areas for TCI and TRI in *R. alpina* and *S. facchinii.*

**Figure 3:**
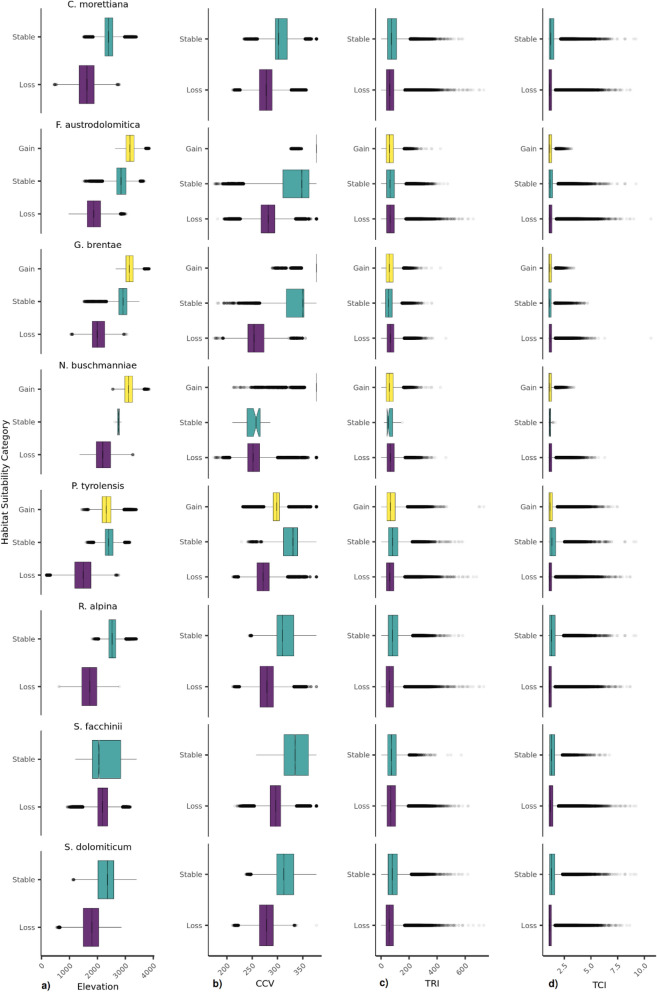
Boxplot for each category of habitat suitability (loss, stable, gain) for the realistic rcp 8.5 scenario for (**a**) elevation (m a.s.l.), (**b**) climate change velocity (log10(m yr^−1^)*100), (**c**) topographical ruggedness index (TRI) and** d** topographical complexity index (TCI) for each of the eight study species. After the pairwise Mann–Whitney tests all the categories showed significant differences, with the following non-significant exceptions for the rcp 8.5 scenario: stable areas compared to lost areas for TCI in *F. austrodolomitica*; stable areas compared to lost and gained areas for both TCI and TRI in *N. buschmanniae.*

For each species, each category of habitat suitability (loss, gain and stability) resulted always significantly different among the different indexes (Tables S5–S6). Similar results were obtained by using randomization, in fact the number of times that* p* values were significant was very high with a minimum of 926 times. Nevertheless, the pairwise test detected the following exceptions that were not significant (Table S7): stable areas compared to and lost areas for TCI in *F. austrodolomitica* for rcp 8.5; stable areas compared to lost and gained areas for both TCI and TRI in *N. buschmanniae*; gained areas compared to lost and stable areas for TCI and TRI for rcp 4.5 in *R. alpina* and *S. facchinii*. In the majority of the species (except for *S. facchinii*), climatically stable areas were expected to occur at high elevation. Also, together with *C. morettiana* and *R. alpina*, *S. facchinii* showed lower values of elevation for the areas of gain in the rcp 4.5 scenario, due to the low extension of gain areas (Table S4). In the rcp 8.5 scenario, the elevational trend was comparable but magnified for all species, compared to the rcp 4.5 scenario for at least the loss/stable comparison, with losses and stability shifted at higher elevations (Figs. 2, 3, Table S7).

Under the rcp 4.5 scenario, TCI had only few times high values in stable areas for six species, while *N. buschmanniae* and *G. brenate* had only few times higher values in lost areas (Fig. 2). The gained areas had low topographic complexity in all the species (except *S. dolomiticum* that had no gain areas). A similar response was detected under the rcp 8.5 scenario but differences were not significant in *F. austrodolomitica* and *N. buschmanniae.* On the contrary*, G. brentae* showed significantly lower TCI values in stable areas (Fig. 3, Supplementary Table S6). The trend of terrain ruggedness (TRI) was similar to that of TCI for four species (*C. morettiana, F. austrodolomitica, N. buschmanniae, P. tyrolensis*), while for *G. brentae* the TRI was only few higher in areas of loss compared to both stable and gained areas (Fig. 3, Supplementary Table S7). Moreover, *R. alpina* did not show significantly higher values in areas of gain. A similar pattern was detected under the rcp 8.5 scenario (Fig. 3, Supplementary Table S7).

Under the rcp 4.5 scenario, six out of eight species (*C. morettiana*, *F. austrodolomitica, G. brentae, N. buschmanniae, P. tyrolensis* and *S. dolomiticum*) had lower values of CCV in lost areas than in stable and gained areas. Differently, in *R. alpina* and *S. facchinii* the lowest values of CCV were recorded in gained areas. In particular, *S. facchinii* was the only species having the highest CCV in the lost areas. Similarly, under the rcp 8.5 scenario, significantly lower values of CCV were recorded in areas expected to be lost by *S. facchinii*. In *N. buschmanniae*, areas projected to be lost and stable had low but not significantly different values of CCV. Moreover, among the species projected to gain range, *F. austrodolomitica, G. brentae* and *N. buschmanniae* showed highest CCV in the areas of gain, while *P. tyrolensis* had lower values of CCV in areas projected to be gained.

## Discussion

This work is the first study that used high resolution SDMs to predict the impact of future climate change in a center of endemism in the Alps (the Dolomites, Italy). Our fine resolution modeling approach is relevant to project the risks of biodiversity loss in mountain systems, where marked microclimatic variation can allow species to persist locally. Further, it is well-suited for the assessment of species-specific climate change threats in particular regions. In line with previous findings in similar parts of the Alps and in other continents^8,9,14,67–69^, our results suggest a likely extensive species-specific reduction of the distributional range of the endemics, little balanced by gain of newly suitable areas. However, the peculiar and complex topography of the Dolomites, where the slope is sharpest at middle elevation, while the declivity decreases forming highlands near the top of the mountains^32,33^, resulted in higher climate change velocity at high elevations and low climate change velocity in middle altitude slopes, where species are projected to lose their suitable habitats.

We projected a reduction in the ranges of high‐altitude endemics of the Dolomites, despite the range contraction strongly depended on the climatic rcp scenario. Despite the fact that all the species were predicted to be negatively affected by climate change, for the subalpine species occurring in the southernmost periphery of the Dolomites (i.e. *P. tyrolensis*), the range loss might be partially buffered by a certain amount of potential gain towards north and towards high altitude. Similarly, high range gain values were detected in the alpine species *G. brentae* and *N. buschmanniae*. Most of the alpine endemics analyzed here occupied only a small part of climatically suitable areas suggesting that post-glacial range-shift limitations may have resulted in a lack of range filling, due to barrier effects and dispersal limitations, as previously detected for other alpine plants^70^. Nevertheless, other factors such as competition, soil conditions and nutrients’ availability could have prevented the range filling^5,71^. However, for these species, a large part of the study area was climatically suitable, and consequently the areas of gain were quantitatively small. Therefore, for these narrow endemic species, the few currently unsuitable areas that became suitable under future climate, were mainly proximal to the currently suitable areas. In contrast, areas currently suitable for *G. brentae* and *N. buschmanniae* occured principally in areas that were occupied extensively by the species. Therefore, considering the poor dispersal capability of endemic species, only *P. tyrolensis* may likely reach the new suitable areas. Differently, the other species that showed a range gain (i.e. *G. brentae* and *N. buschmanniae*), occuring almost only in the Brenta group^41,42^, could hardly keep pace with climate shifts due to the high distance between the current occurrences and the new suitable areas. In line with the hypothesis of a taxon-specific response to climate change, previously detected in Mediterranean mountains^72^, this species-specific response suggested that endemics may respond differently to future climate change, as detected for their response to past climate changes in the Alps^17,73^.

Furthermore, the current global change is predicted to be more extensive than the past changes^4^. The latitudinal and altitudinal shift, which we projected in some endemics, is in line with previous findings on plants both locally and globally^74,75^. Nonetheless, latitudinal and altitudinal shifts to suitable areas may differently affect species survival^76,77^. Indeed, the geographical distance between different climatic zones is shorter along altitudinal than along latitudinal gradients^78^. Consequently, endemics, despite their poor dispersal abilities^79,80^, may more likely keep pace with the upward shift than with the latitudinal one, since lower distances are required to follow the climate shift upward. The eight studied narrow endemic species of the Dolomites have a low dispersal ability (boleochory and blastochory) limited by both the absence of specialized diaspores for dispersal and by the short stem height (Table 1)^26^. Thus, within the time interval of the predicted future climate projections (75 years), all the studied species (except *F. austrodolomitica*) could probably move only a hundred meters away from their current presence (i.e. dispersal distance of about one meter per year), which is very close to a no-dispersal scenario.

According to the general expectation of an upward range shift^35,81^ and the local high‐elevation persistence hypothesis^16^, the majority of endemic species of the Dolomites were projected to lose their range at lowest elevations and to gain (few) or maintain range at highest elevations under both scenarios. Nevertheless, high-altitude endemics like *S. facchinii*, which currently grows above 2600 m a.s.l., and *N. buschmanniae,* with a current range between 2100 to 2400 m a.s.l. only in the Brenta group, could undergo a “no-where to go” scenario only under the rcp 8.5 scenario^82^ (Figures S9–S15).

In the Dolomites, geomorphologically heterogeneous environments were only slightly more complex in areas climatically stable for endemics than in areas where climatic suitability will be lost. Previous studies^18,21,83^ suggested that local geomorphological heterogeneity may increase the availability of microclimatic refugia where alpine species may move or persist^15^. Topographic heterogeneity may cause strong temperatures differences over short distances^15^ and may decouple the local climate from the surrounding landscape buffering the effect of extreme temperatures and favoring survival of relict and endemic species^84^. In endemic centers such as the Dolomites, the rugged topography and resulting microclimatic diversity are thought to have locally buffered the endemics from extinction during past climate change^27,85^. The lower topographic heterogeneity in stable areas detected for *G. brentae* and *N. buschmanniae* is likely explained by the fact that these species were expected to lose all climatically suitable areas within their distributional range and to maintain habitat suitability in the future in northernmost high-altitude flat areas, where topographic complexity was low. The difference in topographic complexity was significant but low between areas of range loss and stability, suggesting that the availability of microclimatic refugia is similar between these areas. Therefore, differences in microrefugia will play a secondary role in assuring endemics survival in the Dolomites.

In general, areas with a slow rate of climate change velocity were expected to provide important refugia for species survival under climate change^28,86^. Contrary to this general expectation, however, we detected that the velocity of climate change was lower in areas where species were projected to lose their suitable habitat than in areas where species were projected to retain or gain suitable habitat. Climate change velocity was inversely related to slope, in fact, the spatial gradient of climatic change was greatest on steep slopes where consequently modest shifts in space are required to meet similar climatic conditions^25^. Although counter-intuitive, the low climate change velocity at low altitudes may be explained by the peculiar topography of the Dolomites, where the slope is sharpest at middle elevation, while the declivity decreases forming highlands near the top of the mountains^87^. The populations of alpine endemics growing at the lowest elevation limit of the species were expected to occur near the species’ warm climatic boundaries^67^. Therefore, they were more prone to extinction risk due to global warming, regardless the low climate change velocity. By contrast, the highlands, where climate change velocity was high, will probably harbor climatic conditions that currently prevail at middle elevations, becoming suitable for endemics in future. In fact, the high elevation areas in the northern and central Dolomites showed the highest values of climate change velocity, making them a difficult terrain for the survival of existing species but possibly also new territory for species projected to occur in these areas under future climate.

### Study limitations

Our results should be considered with particular attention to the main limitations of the approach we adopted. Apart from the use of a different set of GCMs that could provide different results^51^ and the inclusion of missing occurrence records that could affect SDMs^88^, we identified three further main limitations. First, range loss and gain were calculated on binary models’ predictions. A binary cutoff may reduce the predictive ability of models, therefore, such results should not be used to draw inferences on single species but they are useful to infer trends at the level of geographic areas^88^. Second, we modeled the species without any biotic interaction, which has been demonstrated to drive key ecological and evolutionary processes, mediating ecosystem responses to climate change^89^. Third, even if our spatial resolution (50 m) would account for microclimatic variability^90^, small topographical structures under 10 m of resolution like small depressions and crevices could be hardly captured, but they could affect microclimates.

### Conclusive remarks

Our results suggest that alpine narrow endemic plants will be at high risk under climate change in the Alps^9,14^. Topographic and microclimatic conditions may still provide extended areas of refuge only if interventions aimed at reducing and/or offsetting emissions would keep climate change within the rcp 4.5 scenario. Furthermore, the study of the relationship among multiple spatio-temporal predictors (e.g. CCV, TCI etc.) and the species range dynamics might be useful for regional climate-adapted management to avoid overlooking or overestimating the potential impact of climate change locally. The stable areas highlighted in this study are potential “refugia”, areas that should be conservation priorities with the goal of maintaining current patterns of biodiversity. In case of predicted high range loss, the identification of future suitable areas, together with the evaluation of the likelihood of dispersal, are important tools for future conservation planning^91,92^, to maximize the conservation benefit in terms of range loss compensation for rare species at risk of local extinctions.

## Supplementary Information

Supplementary Information.

## Data Availability

We report detailed references of occurrence data in the ‘Supporting information’ file (Table S1). Data on species occurrences as centroid for the 50 m × 50 m cell grid are available at the Figshare data repository 10.6084/m9.figshare.18401966 = data will be made publicly available once the manuscript is accepted for publication.
