# Supplementary material for: Topography of the Dolomites modulates range dynamics of narrow endemic plants under climate change
Source: Sci Rep. 2022 Jan 26;12:1398. doi: 10.1038/s41598-022-05440-3 (PMC8792058; doi:10.1038/s41598-022-05440-3)
Supplement: Supplementary file 1 — Supplementary Information. [file 41598_2022_5440_MOESM1_ESM.docx]

**Topography of the Dolomites modulates range dynamics of narrow endemic plants under climate change**

Francesco Rota^1*^, Gabriele Casazza^2^, Giulio Genova^1^, Gabriele Midolo^1^, Filippo Prosser^3^, Alessio Bertolli^3^, Thomas Wilhalm^4^, Juri Nascimbene^5^, Camilla Wellstein^1^

1 Faculty of Science and Technology, Free University of Bozen‐Bolzano, Bolzano, Italy

2 Dipartimento di Scienze della terra, dell'Ambiente e della Vita, Università di Genova, Corso Europa 26, 16132, Genova, Italy

3 Museo Civico di Rovereto, Rovereto, Trento, Italy

4 Museum of Nature South Tyrol, Via Bottai 1, 39100 Bolzano

5 BIOME Lab, Department of Biological, Geological and Environmental Sciences, Alma Mater Studiorum, University of Bologna, Bologna, Italy.

**Contacts details** (* = corresponding author)

Francesco Rota * ([francesco.rota@natec.unibz.it](mailto:francesco.rota@natec.unibz.it))

ORCID <https://orcid.org/0000-0002-4014-6173>

Gabriele Casazza ([gabriele.casazza@unige.it](mailto:gabriele.casazza@unige.it))

ORCID <https://orcid.org/0000-0002-3334-8551>

Giulio Genova ([giulio.genova@natec.unibz.it](mailto:giulio.genova@natec.unibz.it))

ORCID <https://orcid.org/0000-0001-9412-8651>

Gabriele Midolo (gmidolo@sci.muni.cz)

ORCID <https://orcid.org/0000-0003-1316-2546>

Filippo Prosser ([prosserfilippo@fondazionemcr.it](mailto:prosserfilippo@fondazionemcr.it))

ORCID <https://orcid.org/0000-0002-8723-9860>

Alessio Bertolli ([bertollialessio@fondazionemcr.it](mailto:bertollialessio@fondazionemcr.it))

Thomas Wilhalm ([Thomas.Wilhalm@naturmuseum.it](mailto:Thomas.Wilhalm@naturmuseum.it))

ORCID https://orcid.org/[0000-0001-5871-3562](https://orcid.org/0000-0001-5871-3562)

Juri Nascimbene ([juri.nascimbene@unibo.it](mailto:juri.nascimbene@unibo.it))

ORCID <https://orcid.org/0000-0002-9174-654X>

Camilla Wellstein * ([camilla.wellstein@unibz.it](mailto:camilla.wellstein@unibz.it))

ORCID <https://orcid.org/0000-0001-6994-274X>

**Supplementary information**

**Table S1:** Occurrence points’ source for each province and bibliographic source in the Dolomites

| **area** | **n_occurrences** | **source** |
| --- | --- | --- |
| Provincia Autonoma di Trento | 691 | Prosser et al., 2019, Museo Civico Rovereto |
| Provincia Autonoma di Bolzano/Bozen | 85 | Naturmuseum Südtirol (<http://www.florafauna.it/>), Wilhalm & Hilpold, 2006 |
| Regione Veneto | 136 | Lasen C., Nascimbene J. and Parco Nazionale Dolomiti Bellunesi observations; Da Pozzo et al., 2016 |
| Regione Friuli Venezia-Giulia | 27 | Martini F., Poldini L. and Parco Naturale Dolomiti Friulane observations |

**References:**

Da Pozzo, M., Argenti, C. & Lasen, C. (2016). Atlante floristico delle Dolomiti d’Ampezzo. Specie notevoli, valori ecologici e fitogeografici*. Parco Naturale Regionale delle Dolomiti d’Ampezzo*.

Prosser, F., Bertolli, A., Festi, F. & Perazza, G. (2019). *Flora del Trentino*. Fondazione Museo civico di Rovereto.

Wilhalm, T. & Hilpold, A. *(*2006) Rote Liste der gefährdeten Gefäßpflanzen Südtirols. *Biologisches Landeslabor, Autonome Provinz Bozen, Südtirol, Italien*.

**Table S2:** Modelling framework techniques (CTA – Classification Tree Analysis, GAM – Generalized Additive Models, GBM – Gradient Boosting Machine, GLM – Generalized Linear Models, RF – Random Forest), type (classification tree, regression, machine learning), number of pseudo-absences, number of replicates and method of selection of pseudo-replicates (2°far method, random)

| **modelling technique** | **type** | **number of pseudo-absences** | **number of replicate sets** | **method** |
| --- | --- | --- | --- | --- |
| CTA | classification tree | same as occurrence | 10 | 2°far method |
| GAM | regression | 1000 | 10 | random |
| GLM | regression | 1000 | 10 | random |
| GBM | machine-learning | same as occurrence | 10 | 2°far method |
| RF | machine-learning | same as occurrence | 10 | 2°far method |

**Table S3**: Evaluation values and standard deviations (SD) for each algorithm (CTA – Classification Tree Analysis, GAM – Generalized Additive Models, GBM – Gradient Boosting Machine, GLM – Generalized Linear Models, RF – Random Forest) and species for TSS (true statistical skills) and ROC (receiver operating characteristic or AUC, area under curve)

| **Species** | **Algorithm** | **Eval_met** | **Value (SD)** |
| --- | --- | --- | --- |
| *Campanula morettiana* | CTA | ROC | 0.888(0.038) |
| *Festuca austrodolomitica* | CTA | ROC | 0.92(0.045) |
| *Gentiana brentae* | CTA | ROC | 0.854(0.08) |
| *Nigritella buschmanniae* | CTA | ROC | 0.9(0.043) |
| *Primula tyrolensis* | CTA | ROC | 0.864(0.031) |
| *Rhizobotrya alpina* | CTA | ROC | 0.871(0.027) |
| *Saxifraga facchinii* | CTA | ROC | 0.928(0.042) |
| *Sempervivum dolomiticum* | CTA | ROC | 0.863(0.057) |
| *Campanula morettiana* | CTA | TSS | 0.751(0.059) |
| *Festuca austrodolomitica* | CTA | TSS | 0.802(0.079) |
| *Gentiana brentae* | CTA | TSS | 0.709(0.169) |
| *Nigritella buschmanniae* | CTA | TSS | 0.797(0.082) |
| *Primula tyrolensis* | CTA | TSS | 0.736(0.047) |
| *Rhizobotrya alpina* | CTA | TSS | 0.721(0.052) |
| *Saxifraga facchinii* | CTA | TSS | 0.843(0.086) |
| *Sempervivum dolomiticum* | CTA | TSS | 0.727(0.115) |
| *Campanula morettiana* | GAM | ROC | 0.82(0.02) |
| *Festuca austrodolomitica* | GAM | ROC | 0.928(0.012) |
| *Gentiana brentae* | GAM | ROC | 0.972(0.012) |
| *Nigritella buschmanniae* | GAM | ROC | 0.957(0.016) |
| *Primula tyrolensis* | GAM | ROC | 0.866(0.024) |
| *Rhizobotrya alpina* | GAM | ROC | 0.878(0.024) |
| *Saxifraga facchinii* | GAM | ROC | 0.987(0.004) |
| *Sempervivum dolomiticum* | GAM | ROC | 0.909(0.036) |
| *Campanula morettiana* | GAM | TSS | 0.578(0.035) |
| *Festuca austrodolomitica* | GAM | TSS | 0.811(0.035) |
| *Gentiana brentae* | GAM | TSS | 0.929(0.027) |
| *Nigritella buschmanniae* | GAM | TSS | 0.875(0.026) |
| *Primula tyrolensis* | GAM | TSS | 0.636(0.054) |
| *Rhizobotrya alpina* | GAM | TSS | 0.65(0.053) |
| *Saxifraga facchinii* | GAM | TSS | 0.932(0.026) |
| *Sempervivum dolomiticum* | GAM | TSS | 0.779(0.063) |
| *Campanula morettiana* | GBM | ROC | 0.942(0.018) |
| *Festuca austrodolomitica* | GBM | ROC | 0.961(0.032) |
| *Gentiana brentae* | GBM | ROC | 0.968(0.026) |
| *Nigritella buschmanniae* | GBM | ROC | 0.984(0.008) |
| *Primula tyrolensis* | GBM | ROC | 0.929(0.032) |
| *Rhizobotrya alpina* | GBM | ROC | 0.902(0.026) |
| *Saxifraga facchinii* | GBM | ROC | 0.984(0.017) |
| *Sempervivum dolomiticum* | GBM | ROC | 0.979(0.013) |
| *Campanula morettiana* | GBM | TSS | 0.795(0.063) |
| *Festuca austrodolomitica* | GBM | TSS | 0.833(0.055) |
| *Gentiana brentae* | GBM | TSS | 0.864(0.064) |
| *Nigritella buschmanniae* | GBM | TSS | 0.887(0.037) |
| *Primula tyrolensis* | GBM | TSS | 0.768(0.071) |
| *Rhizobotrya alpina* | GBM | TSS | 0.718(0.048) |
| *Saxifraga facchinii* | GBM | TSS | 0.909(0.026) |
| *Sempervivum dolomiticum* | GBM | TSS | 0.9(0.038) |
| *Campanula morettiana* | GLM | ROC | 0.82(0.01) |
| *Festuca austrodolomitica* | GLM | ROC | 0.935(0.014) |
| *Gentiana brentae* | GLM | ROC | 0.955(0.005) |
| *Nigritella buschmanniae* | GLM | ROC | 0.972(0.006) |
| *Primula tyrolensis* | GLM | ROC | 0.868(0.013) |
| *Rhizobotrya alpina* | GLM | ROC | 0.879(0.033) |
| *Saxifraga facchinii* | GLM | ROC | 0.985(0.004) |
| *Sempervivum dolomiticum* | GLM | ROC | 0.913(0.021) |
| *Campanula morettiana* | GLM | TSS | 0.533(0.031) |
| *Festuca austrodolomitica* | GLM | TSS | 0.828(0.039) |
| *Gentiana brentae* | GLM | TSS | 0.896(0.017) |
| *Nigritella buschmanniae* | GLM | TSS | 0.878(0.021) |
| *Primula tyrolensis* | GLM | TSS | 0.664(0.041) |
| *Rhizobotrya alpina* | GLM | TSS | 0.657(0.076) |
| *Saxifraga facchinii* | GLM | TSS | 0.913(0.031) |
| *Sempervivum dolomiticum* | GLM | TSS | 0.805(0.055) |
| *Campanula morettiana* | RF | ROC | 0.932(0.024) |
| *Festuca austrodolomitica* | RF | ROC | 0.976(0.013) |
| *Gentiana brentae* | RF | ROC | 0.984(0.019) |
| *Nigritella buschmanniae* | RF | ROC | 0.982(0.009) |
| *Primula tyrolensis* | RF | ROC | 0.931(0.02) |
| *Rhizobotrya alpina* | RF | ROC | 0.914(0.031) |
| *Saxifraga facchinii* | RF | ROC | 0.985(0.012) |
| *Sempervivum dolomiticum* | RF | ROC | 0.967(0.035) |
| *Campanula morettiana* | RF | TSS | 0.76(0.062) |
| *Festuca austrodolomitica* | RF | TSS | 0.877(0.047) |
| *Gentiana brentae* | RF | TSS | 0.932(0.049) |
| *Nigritella buschmanniae* | RF | TSS | 0.892(0.053) |
| *Primula tyrolensis* | RF | TSS | 0.754(0.053) |
| *Rhizobotrya alpina* | RF | TSS | 0.732(0.058) |
| *Saxifraga facchinii* | RF | TSS | 0.922(0.037) |
| *Sempervivum dolomiticum* | RF | TSS | 0.887(0.077) |

**Table S4**: Number of pixels and estimated area (1 pixel=50*50 m^2^) for each species and habitat suitability category for the two future projections rcp 4.5 and rcp 8.5 for the climate 2060-2080

| **species** | **Habitat**  **rcp 4.5** | **N°pixels** | **area (km^2^)** | **Habitat**  **rcp 8.5** | **N°pixels** | **area (km^2^)** |
| --- | --- | --- | --- | --- | --- | --- |
| *Campanula morettiana* | Loss | 1121557 | 2803.89 | Loss | 1643775 | 4109.43 |
|  | Gain | 248 | 0.62 | Stable | 238701 | 596.75 |
|  | Stable | 760919 | 1902.30 |  |  |  |
| *Festuca austrodolomitica* | Loss | 906418 | 2266.04 | Loss | 1197987 | 2994.97 |
|  | Gain | 29083 | 72.71 | Gain | 28237 | 70.59 |
|  | Stable | 403655 | 1009.14 | Stable | 112086 | 280.21 |
| *Gentiana brentae* | Loss | 114447 | 286.12 | Loss | 164736 | 411.84 |
|  | Gain | 53610 | 134.02 | Gain | 46149 | 115.37 |
|  | Stable | 94307 | 235.77 | Stable | 44018 | 110.04 |
| *Nigritella buschmanniae* | Loss | 110969 | 277.43 | Loss | 120656 | 301.64 |
|  | Gain | 72218 | 180.54 | Gain | 55445 | 138.61 |
|  | Stable | 9744 | 24.36 | Stable | 57 | 0.14 |
| *Primula tyrolensis* | Loss | 1103110 | 2757.77 | Loss | 1597491 | 3993.73 |
|  | Gain | 426494 | 1066.23 | Gain | 214453 | 536.13 |
|  | Stable | 575417 | 1438.54 | Stable | 81036 | 202.59 |
| *Rhizobotrya alpina* | Loss | 994113 | 2485.28 | Loss | 1412640 | 3531.60 |
|  | Gain | 9 | 0.02 | Stable | 133562 | 333.90 |
|  | Stable | 552089 | 1380.22 |  |  |  |
| *Saxifraga facchinii* | Loss | 388591 | 971.48 | Loss | 524056 | 1310.14 |
|  | Gain | 31 | 0.08 | Stable | 13577 | 33.94 |
|  | Stable | 149042 | 372.60 |  |  |  |
| *Sempervivum dolomiticum* | Loss | 538263 | 1345.68 | Loss | 1065200 | 2663 |
|  | Stable | 714833 | 1787.08 | Stable | 187896 | 469.74 |

**Table S5-S6.** We tested for each species and each scenario (rcp 4.5 and rcp 8.5), based on the Kruskal-Wallis test, the significance of the relationship of habitat suitability with each of the indexes: climate change velocity (log_10_(m yr^-1^)*100), elevation (m a.s.l.), topographical complexity index (TCI), topographical ruggedness index (TRI)

|  | **~ Habitat suitability Rcp 4.5** | | | | | | | | | | | |
| --- | --- | --- | --- | --- | --- | --- | --- | --- | --- | --- | --- | --- |
|  | **CCV_45** | | | **elevation** | | | **TRI** | | | **TCI** | | |
| **species** | **Chi-squared** | **df** | **p.value** | **Chi-squared** | **df** | **p.value** | **Chi-squared** | **df** | **p.value** | **Chi-squared** | **df** | **p.value** |
| *Campanula morettiana* | 367341.57 | 2 | <0.05 | 1196977.97 | 2 | <0.05 | 3613.12 | 2 | <0.05 | 5413.04 | 2 | <0.05 |
| *Festuca austrodolomitica* | 88748.84 | 2 | <0.05 | 565631.26 | 2 | <0.05 | 9572.02 | 2 | <0.05 | 17970.17 | 2 | <0.05 |
| *Gentiana brentae* | 28330.22 | 2 | <0.05 | 161404.94 | 2 | <0.05 | 985.92 | 2 | <0.05 | 1751.54 | 2 | <0.05 |
| *Nigritella buschmanniae* | 25200.95 | 2 | <0.05 | 127997.02 | 2 | <0.05 | 4921.10 | 2 | <0.05 | 6917.90 | 2 | <0.05 |
| *Primula tyrolensis* | 699037.95 | 2 | <0.05 | 1366858.67 | 2 | <0.05 | 3208.45 | 2 | <0.05 | 9863.08 | 2 | <0.05 |
| *Rhizobotrya alpina* | 164904.89 | 2 | <0.05 | 865438.73 | 2 | <0.05 | 30688.81 | 2 | <0.05 | 39756.76 | 2 | <0.05 |
| *Saxifraga facchinii* | 13715.68 | 2 | <0.05 | 212.51 | 2 | <0.05 | 5263.73 | 2 | <0.05 | 15611.95 | 2 | <0.05 |
| *Sempervivum dolomiticum* | 302.99 | 1 | <0.05 | 85153.45 | 1 | <0.05 | 16040.28 | 1 | <0.05 | 31047.45 | 1 | <0.05 |

|  | **~ Habitat suitability Rcp 8.5** | | | | | | | | | | | |
| --- | --- | --- | --- | --- | --- | --- | --- | --- | --- | --- | --- | --- |
|  | **CCV_85** | | | **elevation** | | | **TRI** | | | **TCI** | | |
| **species** | **Chi-squared** | **df** | **p.value** | **Chi-squared** | **df** | **p.value** | **Chi-squared** | **df** | **p.value** | **Chi-squared** | **df** | **p.value** |
| *Campanula morettiana* | 363536.07 | 1 | <0.05 | 582750.69 | 1 | <0.05 | 16230.99 | 1 | <0.05 | 22679.98 | 1 | <0.05 |
| *Festuca austrodolomitica* | 222176.96 | 2 | <0.05 | 358276.75 | 2 | <0.05 | 260.19 | 2 | <0.05 | 1286.98 | 2 | <0.05 |
| *Gentiana brentae* | 166164.52 | 2 | <0.05 | 166498.20 | 2 | <0.05 | 4042.16 | 2 | <0.05 | 5342.20 | 2 | <0.05 |
| *Nigritella buschmanniae* | 110154.09 | 2 | <0.05 | 109208.98 | 2 | <0.05 | 1050.96 | 2 | <0.05 | 1825.14 | 2 | <0.05 |
| *Primula tyrolensis* | 523769.3 | 2 | <0.05 | 695071.38 | 2 | <0.05 | 17072.35 | 2 | <0.05 | 27835.06 | 2 | <0.05 |
| *Rhizobotrya alpina* | 242728.84 | 1 | <0.05 | 351627.71 | 1 | <0.05 | 27603.04 | 1 | <0.05 | 37020.90 | 1 | <0.05 |
| *Saxifraga facchinii* | 16841.61 | 1 | <0.05 | 151.67 | 1 | <0.05 | 174.62 | 1 | <0.05 | 1404.87 | 1 | <0.05 |
| *Sempervivum dolomiticum* | 290673.46 | 1 | <0.05 | 211943.06 | 1 | <0.05 | 32015.71 | 1 | <0.05 | 52641.76 | 1 | <0.05 |

**Table S7.** For each index in each scenario, we tested for significant differences in the index expression for the areas of habitat suitability loss, stability and gain. The letters refer to the p-values’ comparison after a Mann-Whitney test with significance at 0.05 with Bonferroni correction for each category of habitat suitability (loss, stable, gain), for each rcp 4.5 and rcp 8.5 scenario, for climate change velocity (log_10_(m yr^-1^)*100), elevation (m a.s.l.), topographical complexity index (TCI), topographical ruggedness index (TRI), for each of the eight study species.

| **species** | **rcp4.5** | **CCV_45** | **elevation** | **TCI** | **TRI** | **rcp8.5** | **CCV_85** | **elevation** | **TCI** | **TRI** |
| --- | --- | --- | --- | --- | --- | --- | --- | --- | --- | --- |
| *Campanula morettiana* | Loss | a | a | a | a | Loss | a | a | a | a |
|  | Stable | b | b | b | b | Stable | b | b | b | b |
|  | Gain | c | c | c | c |  |  |  |  |  |
| *Festuca austrodolomitica* | Loss | a | a | a | a | Loss | a | a | a | a |
|  | Stable | b | b | b | b | Stable | b | b | a | b |
|  | Gain | c | c | c | c | Gain | c | c | b | c |
| *Gentiana brentae* | Loss | a | a | a | a | Loss | a | a | a | a |
|  | Stable | b | b | b | b | Stable | b | b | b | b |
|  | Gain | c | c | c | c | Gain | c | c | c | c |
| *Nigritella buschmanniae* | Loss | a | a | a | a | Loss | a | a | a | a |
|  | Stable | b | b | b | b | Stable | a | b | ab | ab |
|  | Gain | b | c | c | c | Gain | b | c | b | b |
| *Primula tyrolensis* | Loss | a | a | a | a | Loss | a | a | a | a |
|  | Stable | b | b | b | b | Stable | b | b | b | b |
|  | Gain | c | c | c | c | Gain | c | c | c | c |
| *Rhizobotrya alpina* | Loss | a | a | a | a | Loss | a | a | a | a |
|  | Stable | b | b | b | b | Stable | b | b | b | b |
|  | Gain | c | c | ab | ab |  |  |  |  |  |
| *Saxifraga facchinii* | Loss | a | a | a | a | Loss | a | a | a | a |
|  | Stable | b | b | b | b | Stable | b | b | b | b |
|  | Gain | c | c | ab | ab |  |  |  |  |  |
| *Sempervivum dolomiticum* | Loss | a | a | a | a | Loss | a | a | a | a |
|  | Stable | b | b | b | b | Stable | b | b | b | b |


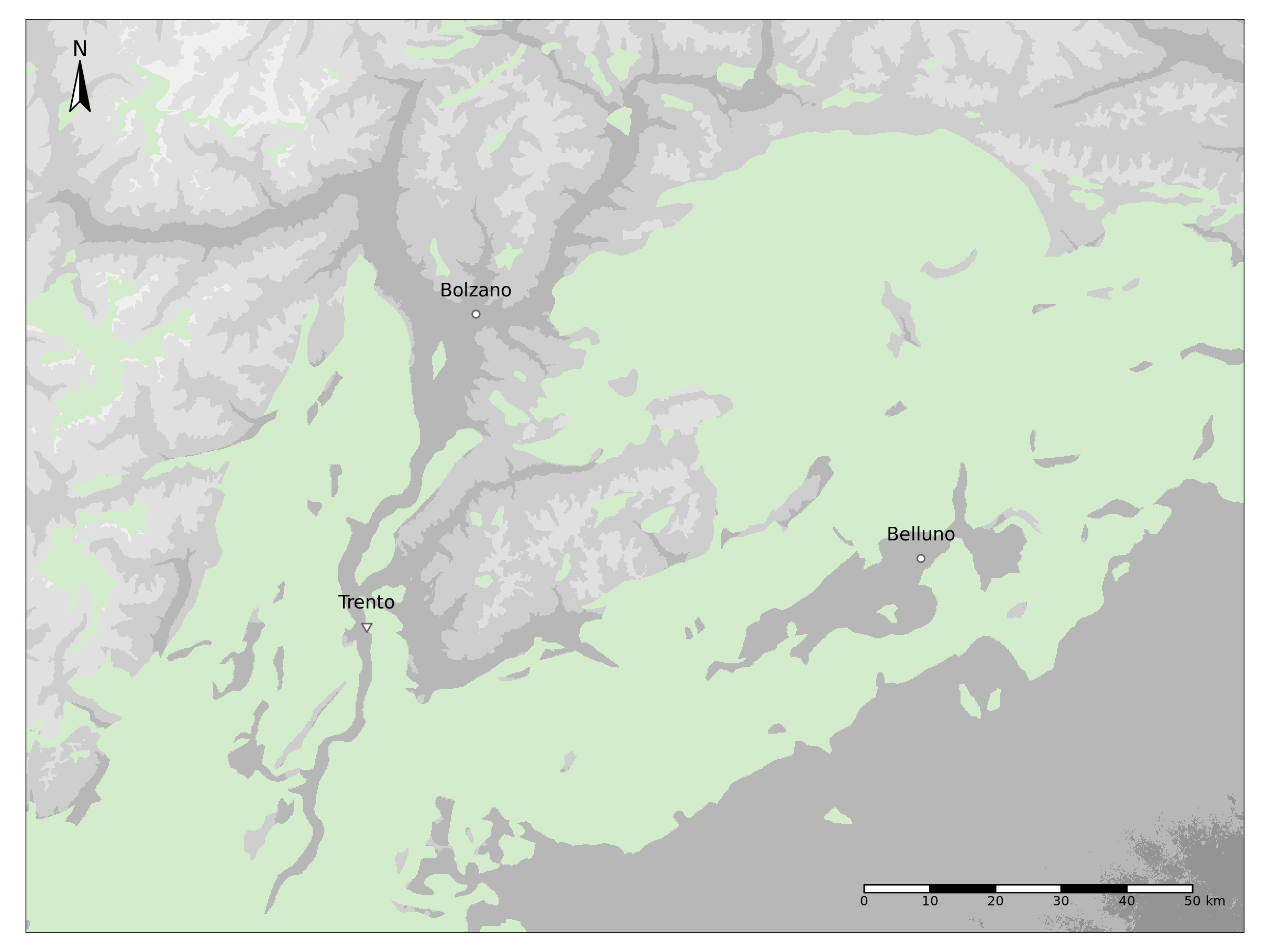


**Figure S1**: The calcareous and dolomitic bedrock in the study area is shown in green color


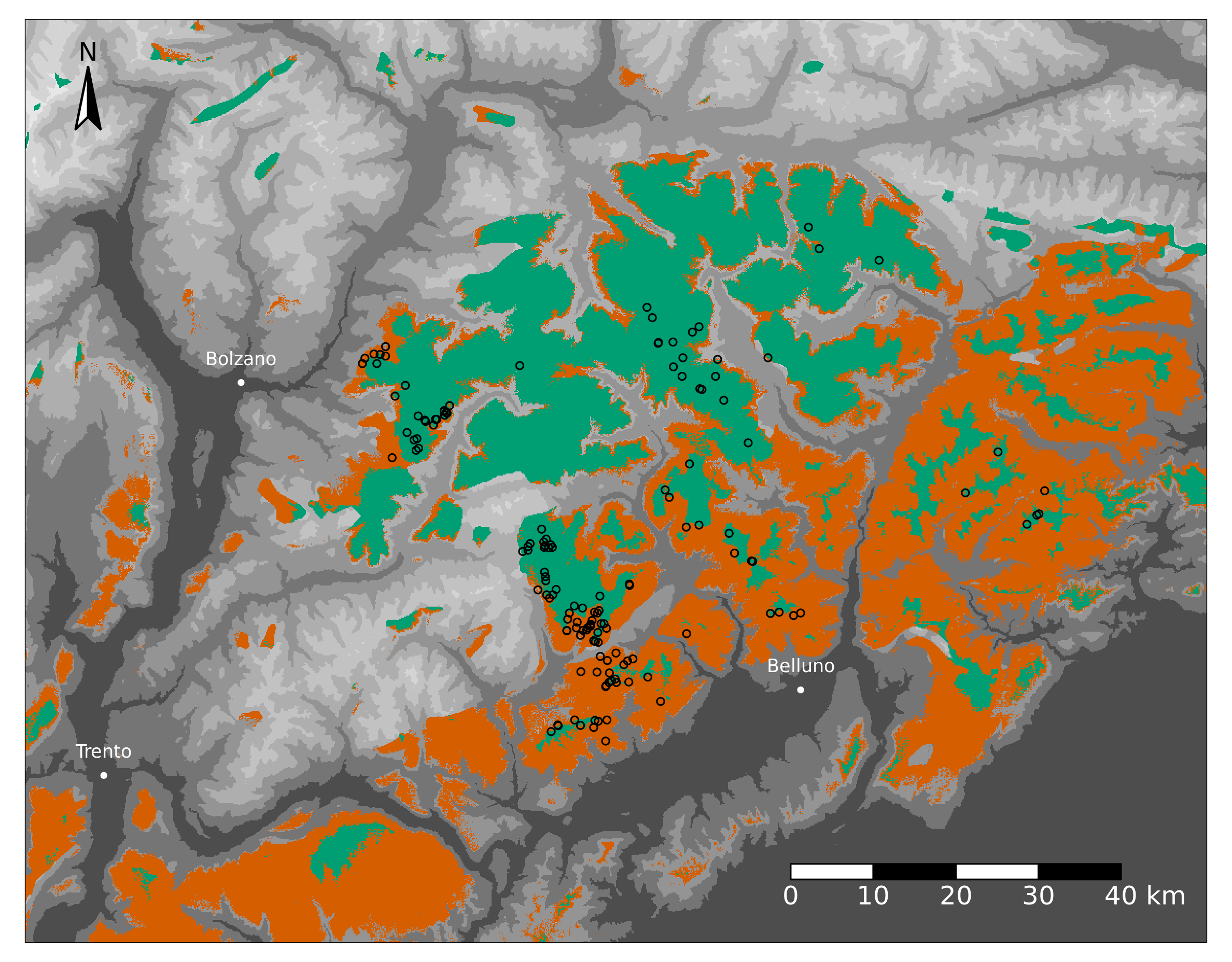
**Figure S2**: *Campanula morettiana* prediction map for the intermediate scenario (rcp 4.5). Stable areas are shown in green, areas of loss are shown in orange, gain areas are few and not visible at this size. The circles represent the occurrence points.


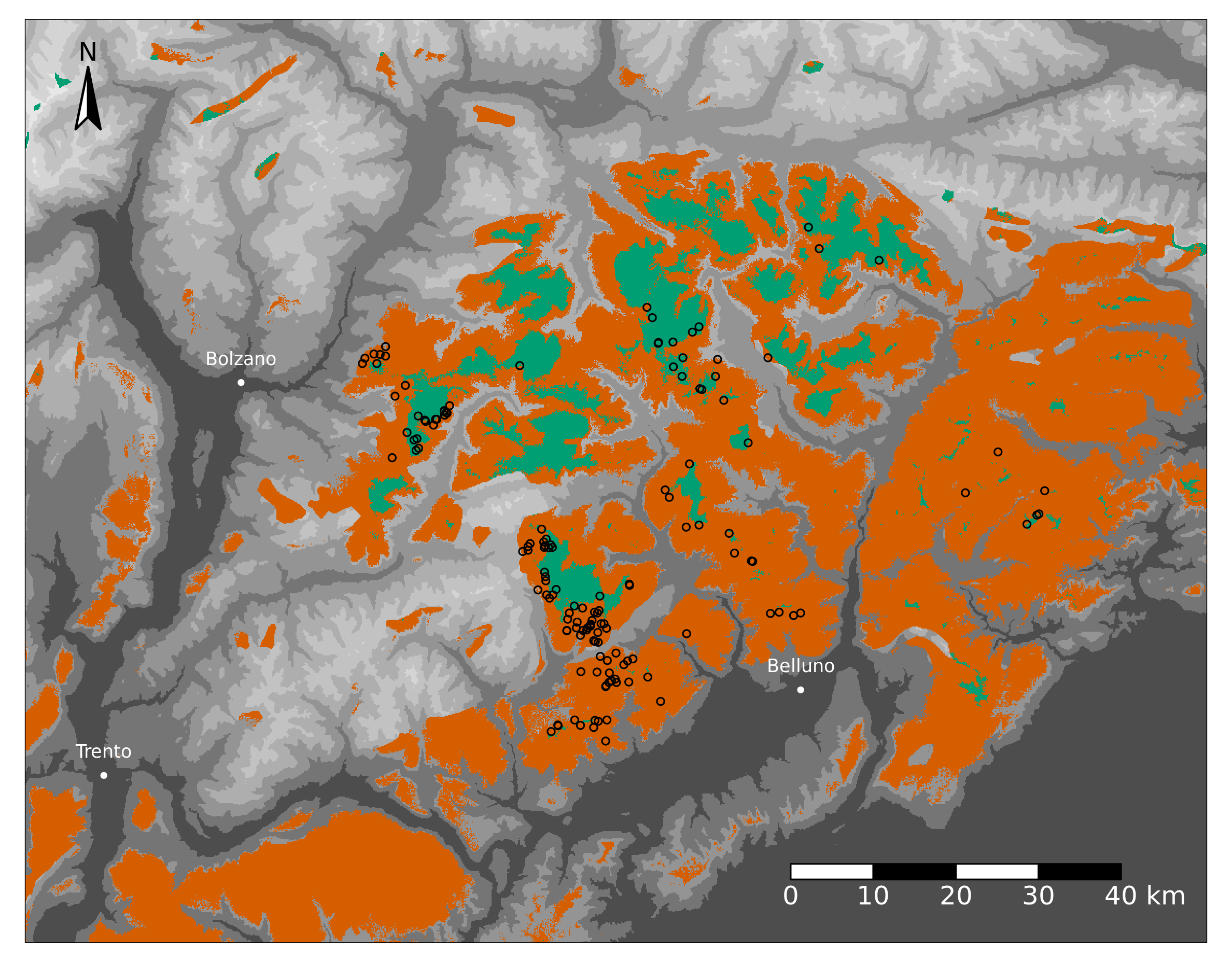
 **Figure S3**: *Campanula morettiana* prediction map for the realistic scenario (rcp 8.5). Stable areas are shown in green, areas of loss are shown in orange, gain areas are not present. The circles represent the occurrence points.


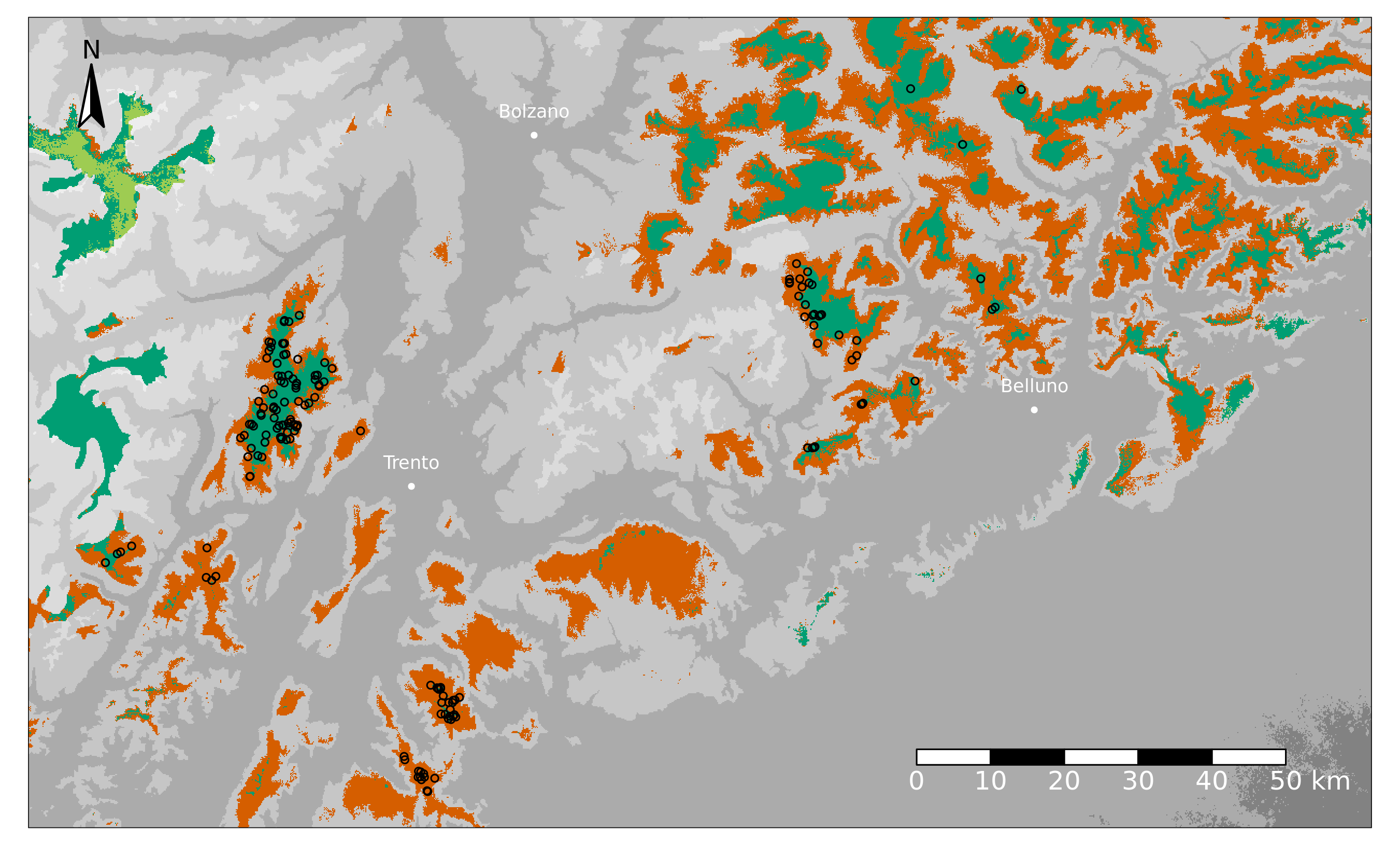
 **Figure S4**: *Festuca austrodolomitica* prediction map for the intermediate scenario (rcp 4.5). Stable areas are shown in green, areas of loss are shown in orange, areas of gain of climatic habitat suitability are shown in light green, the circles represent the occurrence points


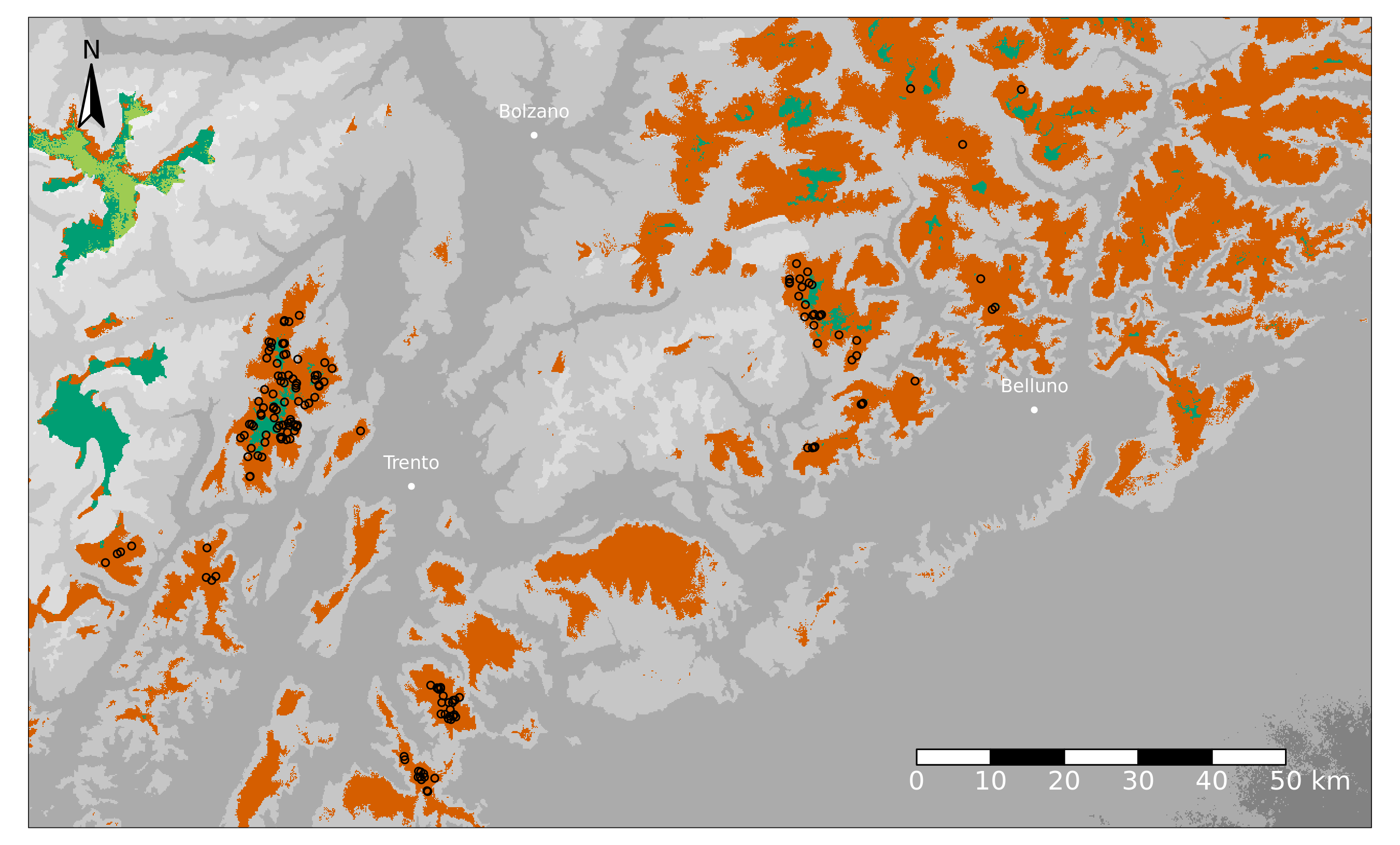
 **Figure S5**: *Festuca austrodolomitica* prediction map for the realistic scenario (rcp 8.5). Stable areas are shown in green, areas of loss are shown in orange, areas of gain of climatic habitat suitability are shown in light green, the circles represent the occurrence points


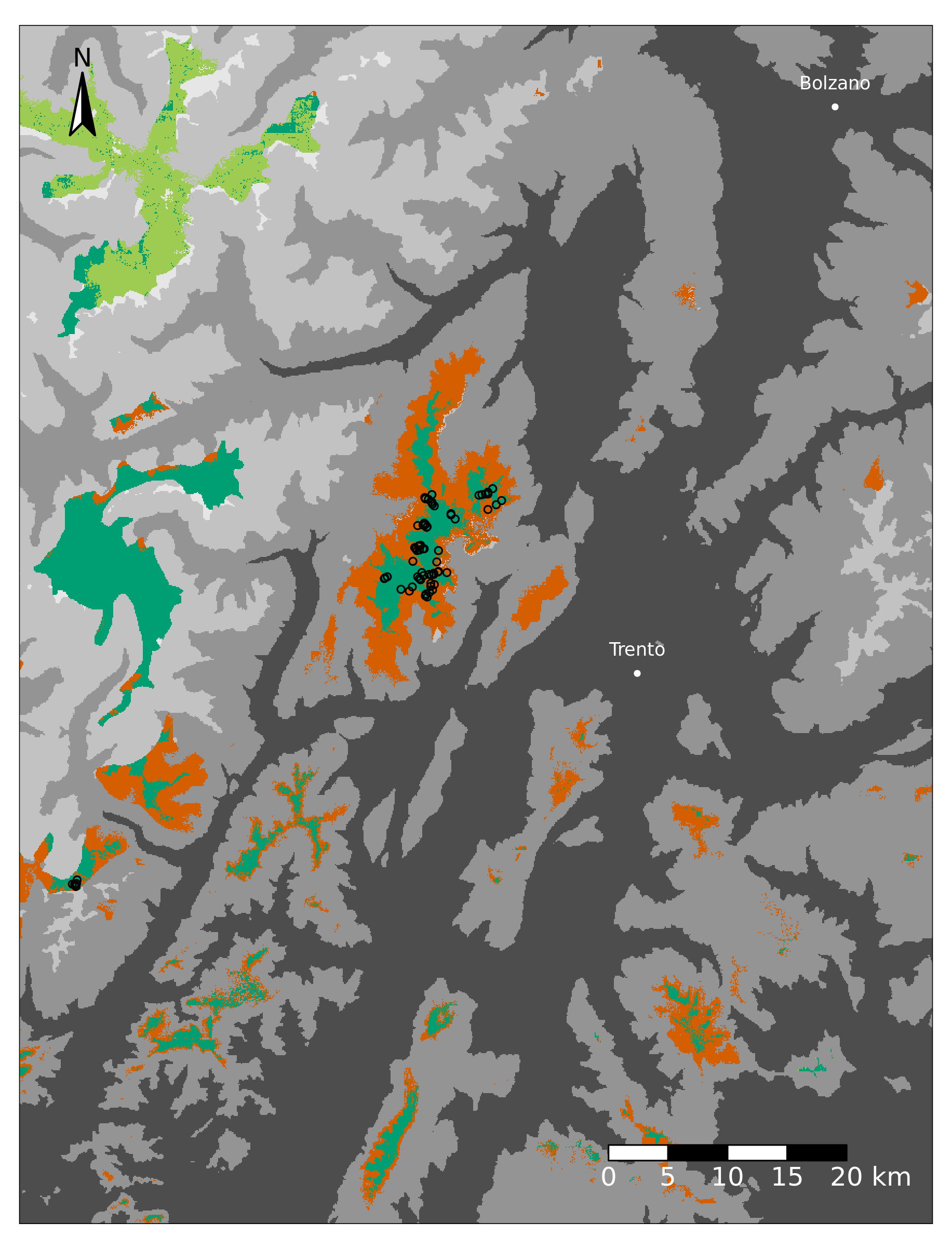
 **Figure S6***: Gentiana brentae* prediction map for the intermediate scenario (rcp 4.5). Stable areas are shown in green, areas of loss are shown in orange, areas of gain of climatic habitat suitability are shown in light green, the circles represent the occurrence points


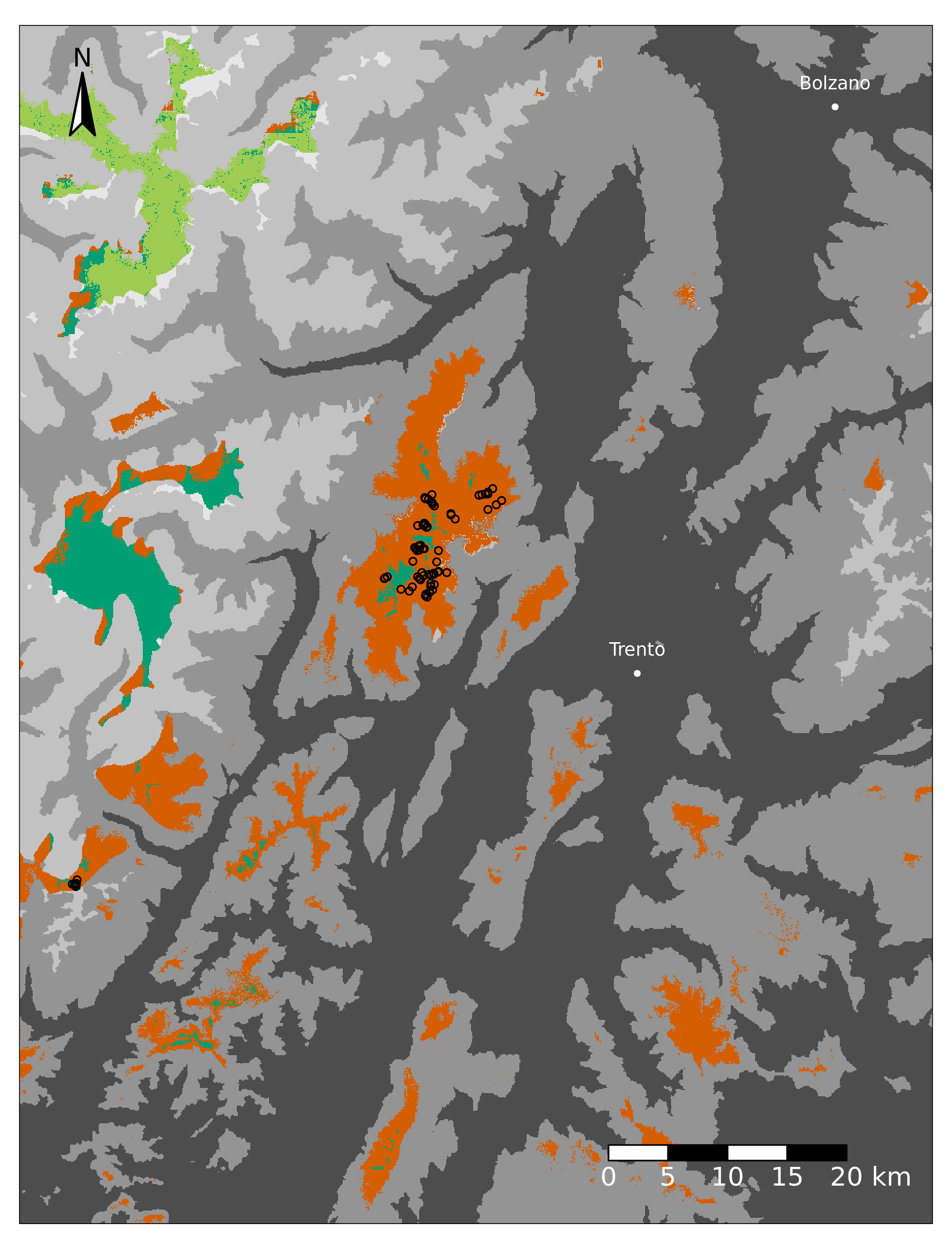
 **Figure S7**: *Gentiana brentae* prediction map for the realistic scenario (rcp 8.5). Stable areas are shown in green, areas of loss are shown in orange, areas of gain of climatic habitat suitability are shown in light green, the circles represent the occurrence points


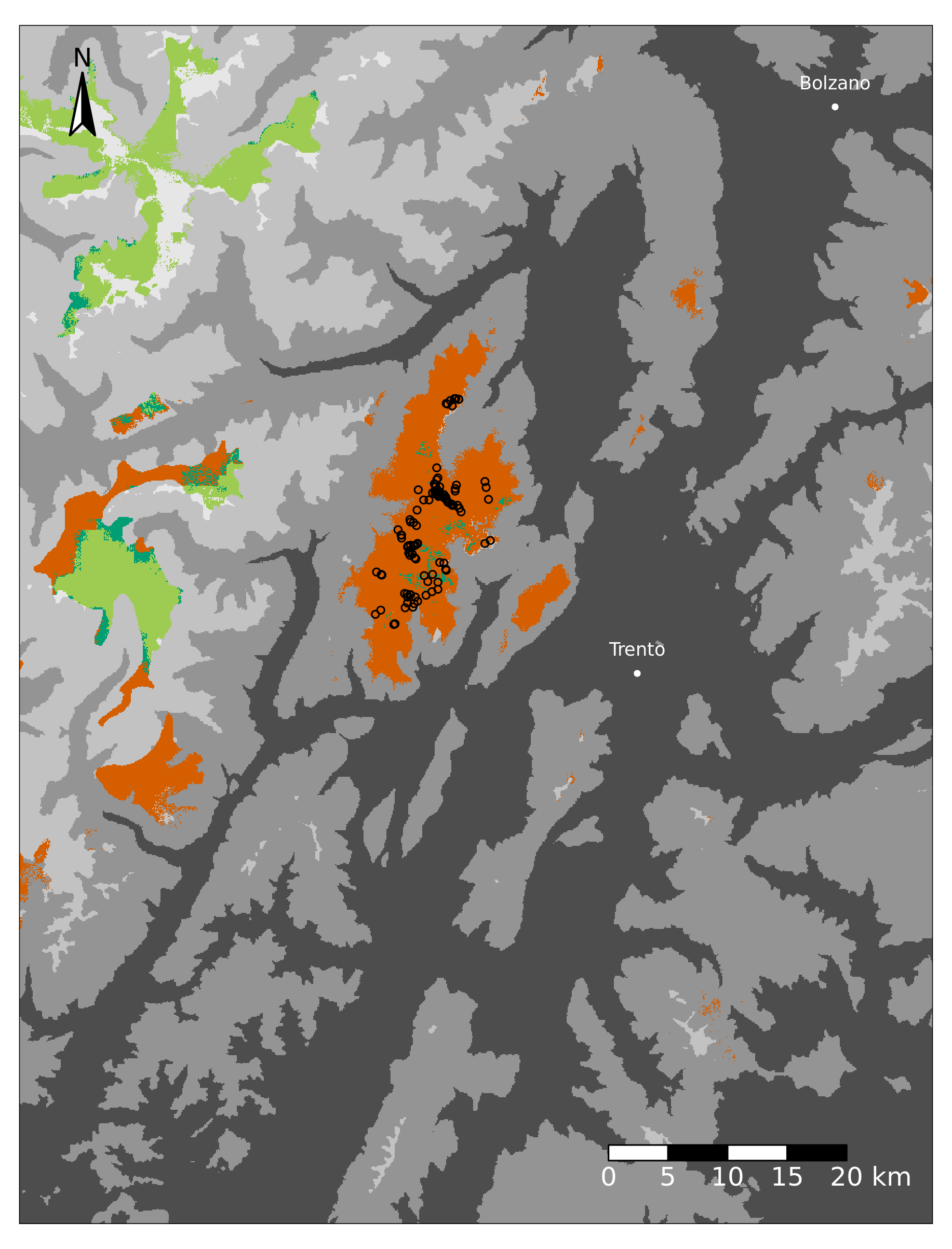
 **Figure S8**: *Nigritella buschmanniae* prediction map for the intermediate scenario (rcp 4.5). Stable areas are shown in green, areas of loss are shown in orange, areas of gain of climatic habitat suitability are shown in light green, the circles represent the occurrence points


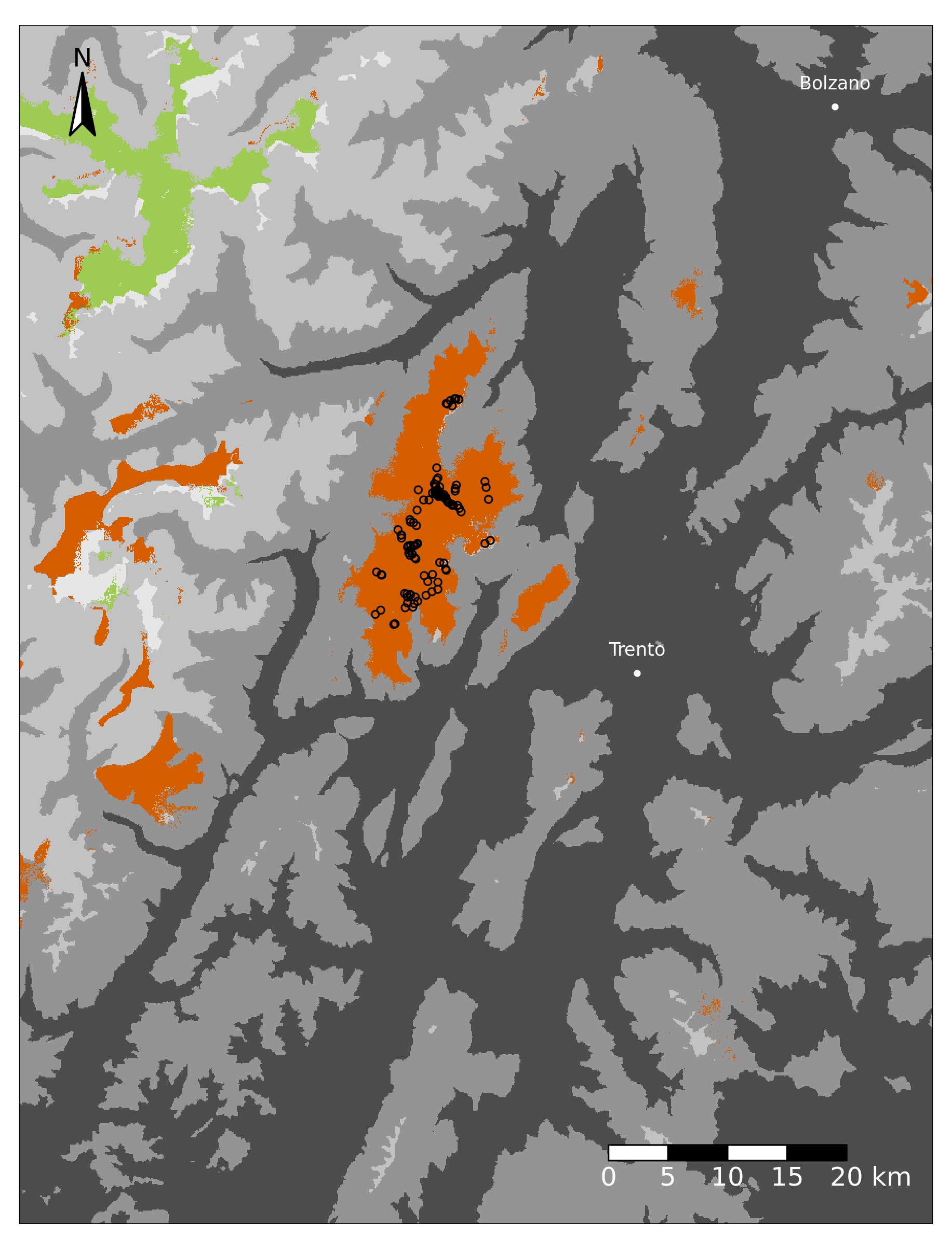
 **Figure S9**: *Nigritella buschmanniae* prediction map for the realistic scenario (rcp 8.5). Stable areas were few and not visible, areas of loss are shown in orange, areas of gain of climatic habitat suitability are shown in light green, the circles represent the occurrence points


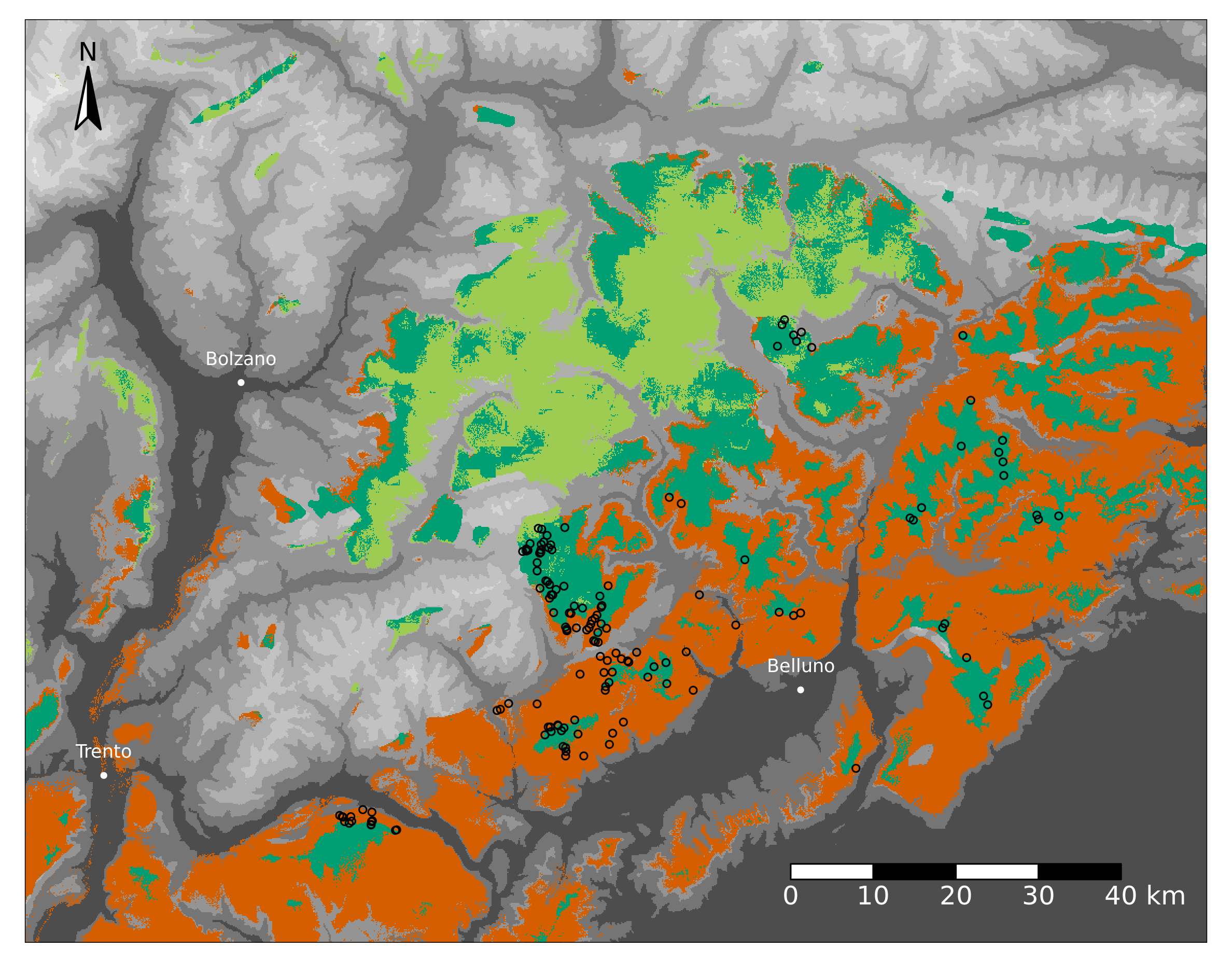
 **Figure S10**: *Primula tyrolensis* prediction map for the intermediate scenario (rcp 4.5). Stable areas are shown in green, areas of loss are shown in orange, areas of gain of climatic habitat suitability are shown in light green, the circles represent the occurrence points


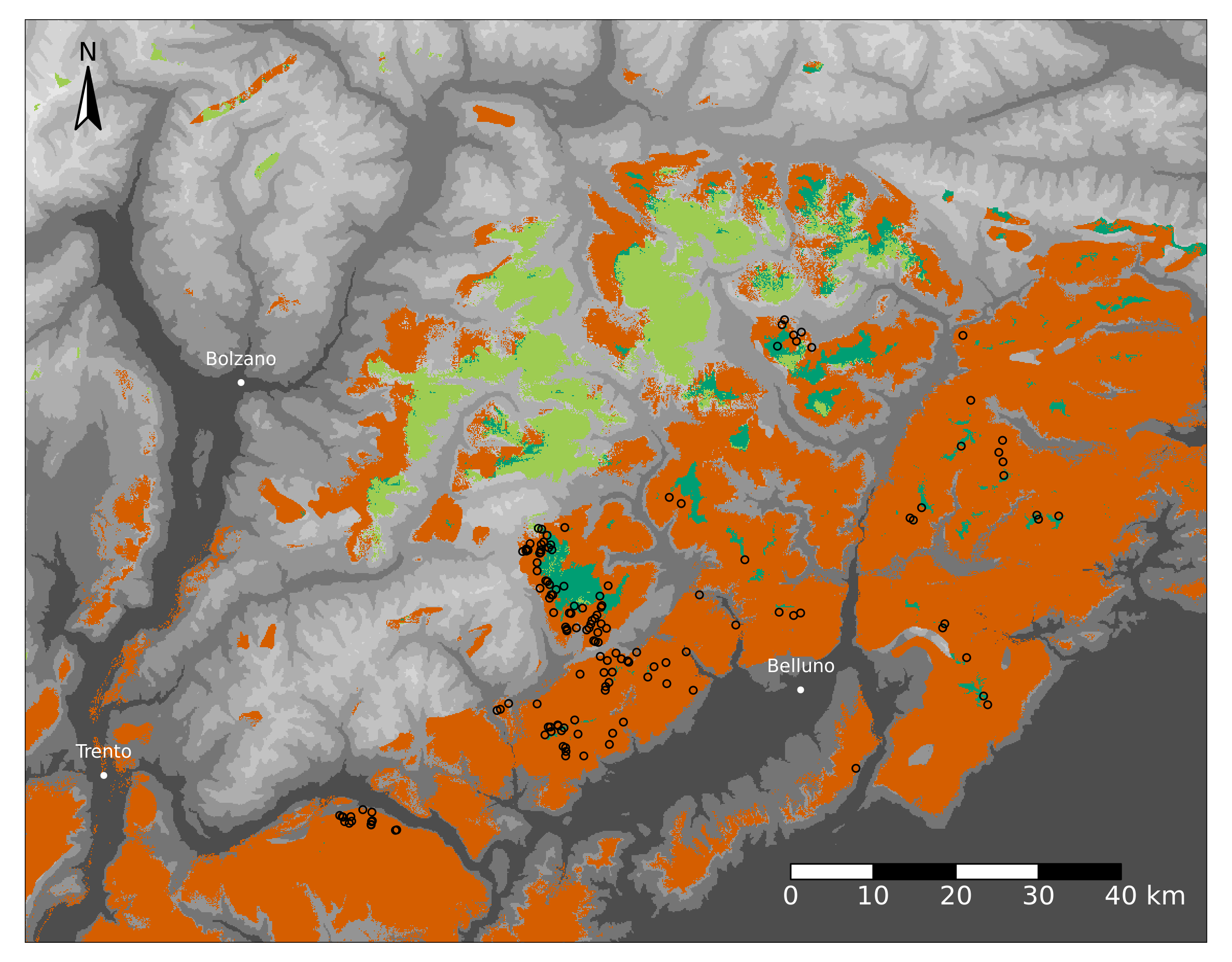
 **Figure S11**: *Primula tyrolensis* prediction map for the realistic scenario (rcp 8.5). Stable areas are shown in green, areas of loss are shown in orange, areas of gain of climatic habitat suitability are shown in light green, the circles represent the occurrence points


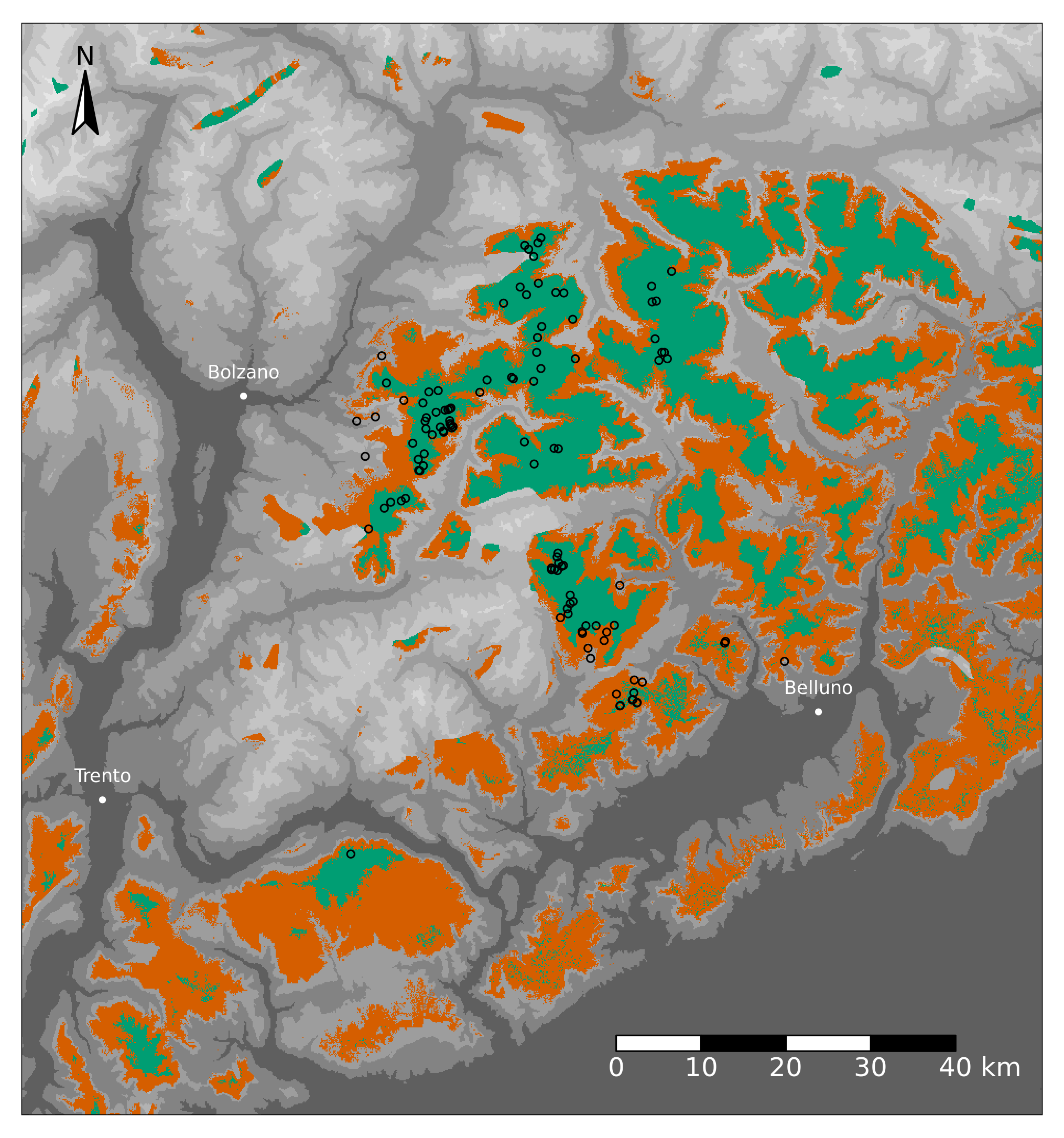
 **Figure S12**: *Rhizobotrya alpina* prediction map for the intermediate scenario (rcp 4.5). Stable areas are shown in green, areas of loss are shown in orange, areas of gain of climatic habitat suitability are few and not visible at this size. The circles represent the occurrence points.


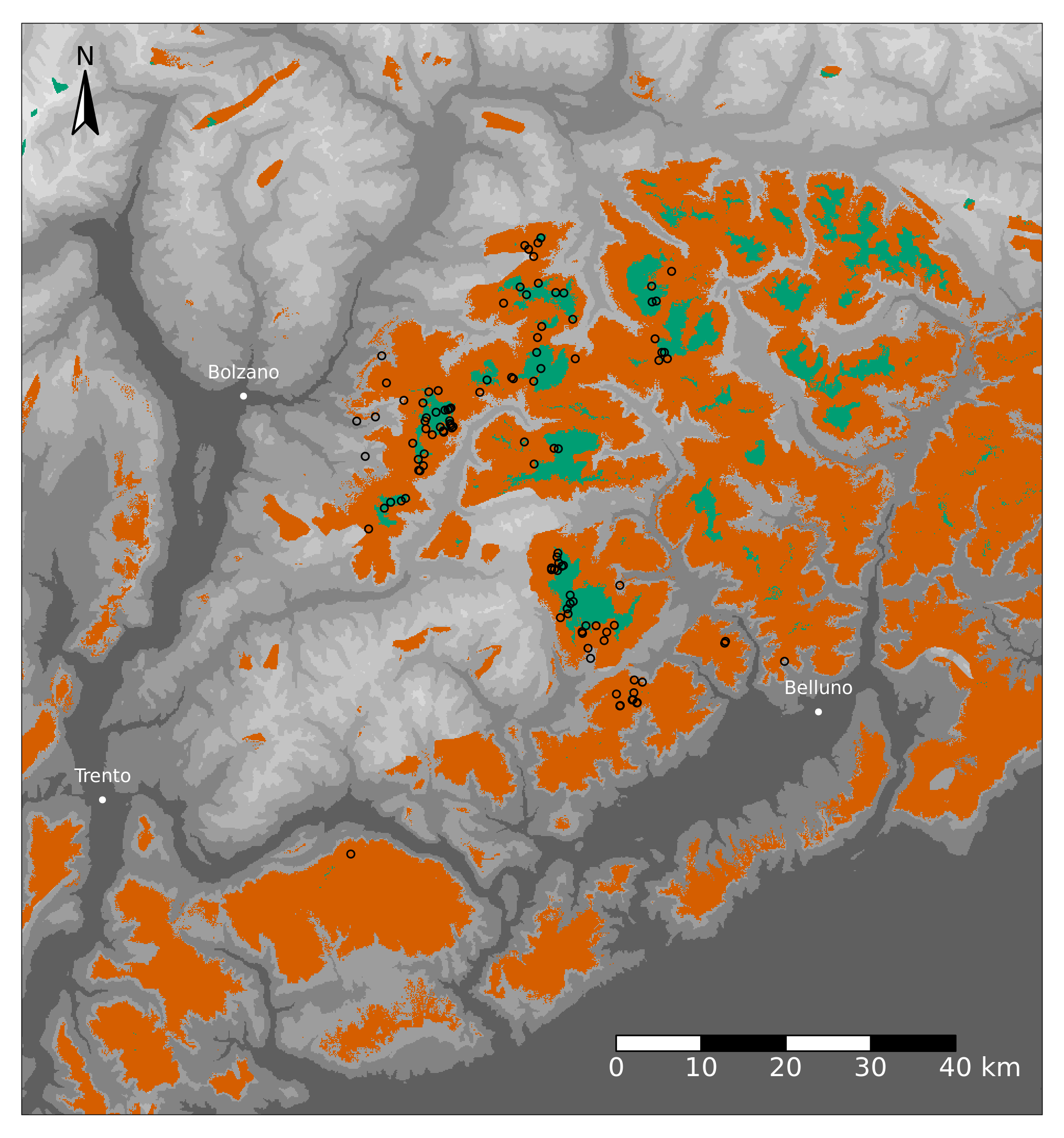
 **Figure S13**: *Rhizobotrya alpina* prediction map for the realistic scenario (rcp 8.5). Stable areas are shown in green, areas of loss are shown in orange, gain areas not present. The circles represent the occurrence points.


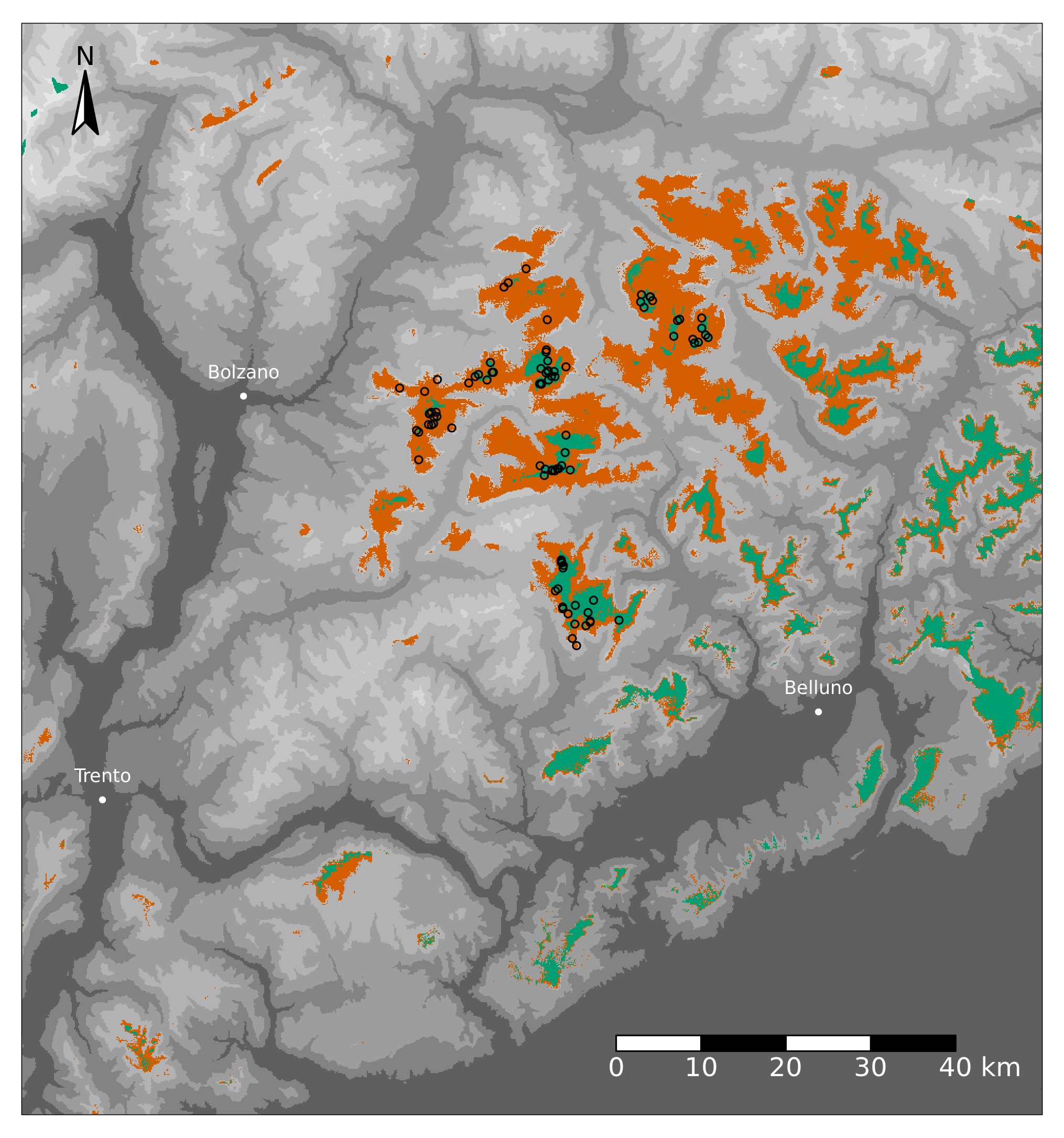
 **Figure S14**: *Saxifraga facchinii* prediction map for the intermediate scenario (rcp 4.5). Stable areas are shown in green, areas of loss are shown in orange, areas of gain of climatic habitat suitability are few and not visible at this size. The circles represent the occurrence points.


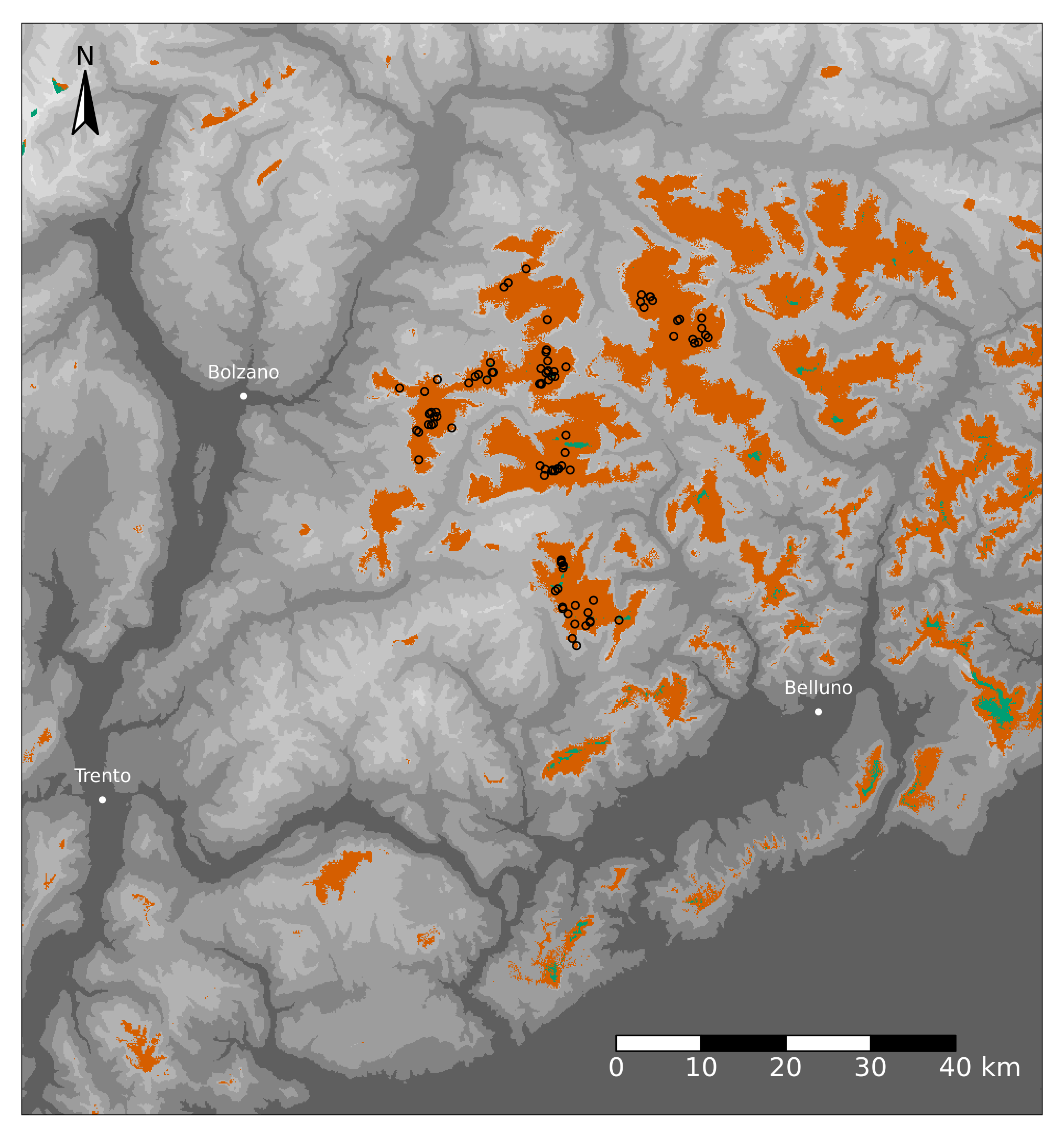
 **Figure S15**: *Saxifraga facchinii* prediction map for the realistic scenario (rcp 8.5). Stable areas are shown in green, areas of loss are shown in orange, areas of gain of climatic habitat suitability are not present. The circles represent the occurrence points.


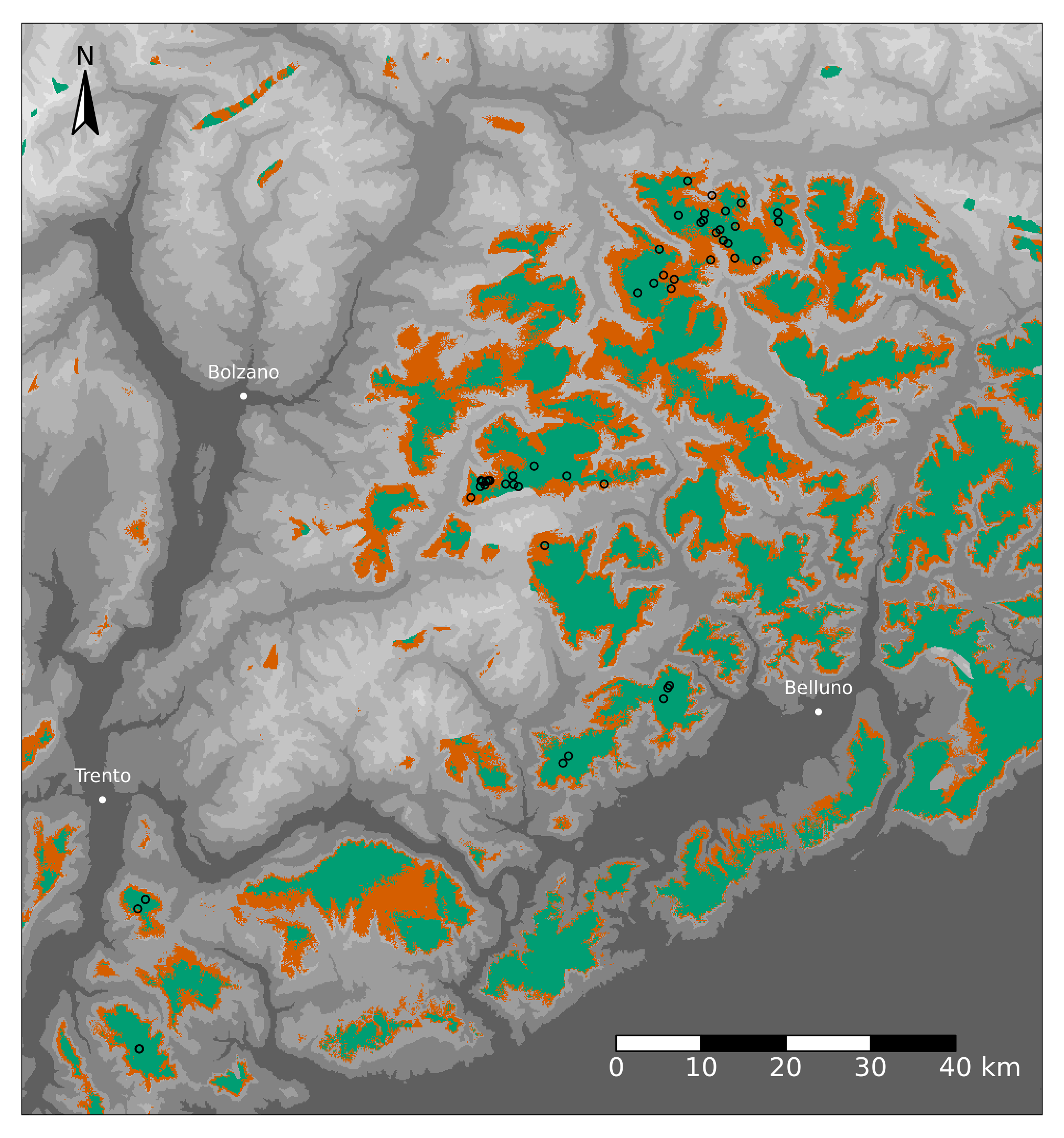
 **Figure S16**: *Sempervivum dolomiticum* prediction map for the intermediate scenario (rcp 4.5). Stable areas are shown in green, areas of loss are shown in orange, areas of gain of climatic habitat suitability are not present. The circles represent the occurrence points.


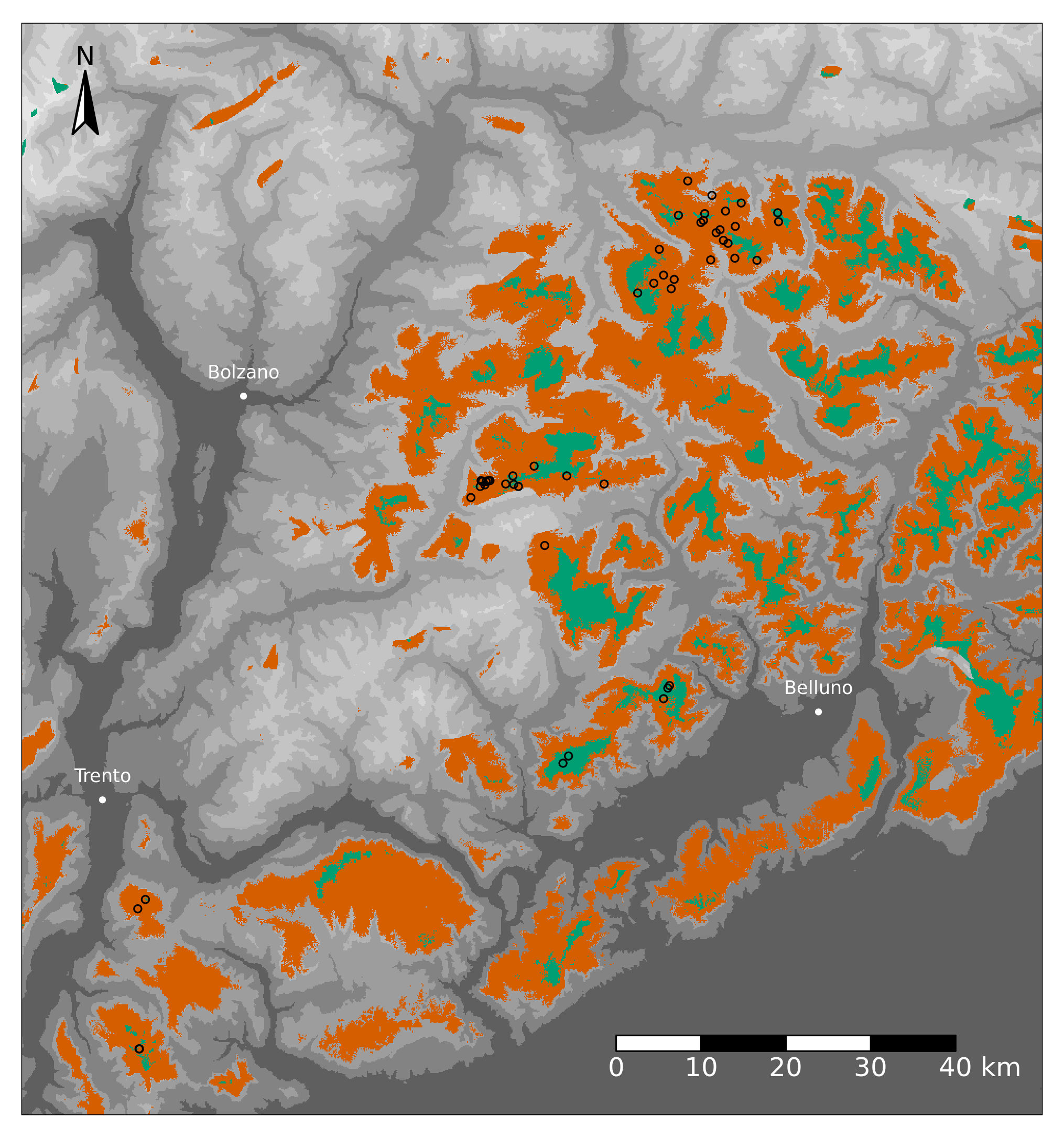
 **Figure S17**: *Sempervivum dolomiticum* prediction map for the realistic scenario (rcp 8.5). Stable areas are shown in green, areas of loss are shown in orange, areas of gain of climatic habitat suitability are not present. The circles represent the occurrence points
